# The role of phenylalanine and tyrosine in longevity: a cohort and Mendelian randomization study

**DOI:** 10.18632/aging.206326

**Published:** 2025-10-03

**Authors:** Jie V. Zhao, Yitang Sun, Junmeng Zhang, Kaixiong Ye

**Affiliations:** 1 School of Public Health, Li Ka Shing Faculty of Medicine, The University of Hong Kong, Hong Kong SAR, China; 2 State Key Laboratory of Pharmaceutical Biotechnology, The University of Hong Kong, Hong Kong SAR, China; 3 Department of Genetics, University of Georgia, Athens, Georgia, GA 30602, USA; 4 Institute of Bioinformatics, University of Georgia, Athens, Georgia, GA 30602, USA

**Keywords:** lifespan, phenylalanine, tyrosine, mendelian randomization, sex-specific

## Abstract

Background: Protein restriction increases lifespan, however, the specific amino acids affecting lifespan are unclear. Tyrosine and its precursor, phenylalanine, may influence lifespan through their response to low-protein diet, with possible sex disparity.

Methods: We applied cohort study design and Mendelian randomization (MR) analysis. Specifically, we examined the overall and sex-specific relationships between circulating phenylalanine and tyrosine and all-cause mortality in the UK Biobank using Cox regression. To test causality, in two-sample MR analysis, we used genetic variants associated with phenylalanine and tyrosine in UK Biobank with genome-wide significance and uncorrelated (r^2^ < 0.001) with each other, and applied them to large genome-wide association studies of lifespan, including parental, paternal, and maternal attained ages in the UK Biobank. We also conducted multivariable MR to examine the independent role of phenylalanine and tyrosine.

Results: Tyrosine was associated with shorter lifespan in both observational and MR study, with potential sex disparity. After controlling for phenylalanine using multivariable MR, tyrosine remained related to a shorter lifespan in men (−0.91 years of life, 95% confidence interval (CI) −1.60 to −0.21) but not in women (−0.36 years, 95% CI −0.96 to 0.23). Phenylalanine showed no association with lifespan in either men or women after controlling for tyrosine.

Conclusions: Reducing tyrosine in people with elevated concentrations may contribute to prolonging lifespan, with potential sex-specific differences. It is worthwhile to explore pathways underlying the sex-specific effects.

## INTRODUCTION

Enhancing longevity and living a healthy life at older age are key objectives for healthcare systems worldwide. Dietary protein intake regulates longevity across various species [1]. Protein restriction has also been demonstrated to extend lifespan [2]. As proteins are composed of amino acids, we hypothesize that amino acids responding to the effects of protein restriction may affect lifespan. In an animal experiment, tyrosine has been shown to be specifically involved in regulation of the physiological response to low-protein diet [1]. Another animal experiment further shows that restriction of tyrosine intake lowers internal tyrosine levels, modulates amino acid-sensing pathways, and prolongs lifespan [3]. Tyrosine plays a critical role in metabolic pathways as a precursor to important neurotransmitters like dopamine, norepinephrine, and epinephrine [4]. These neurotransmitters are crucial for regulating mood, cognition, and stress responses [5], which are vital for metabolic health and potentially influencing lifespan [6]. Tyrosine deprivation may also lead to suppression of IIS and mTORC1 pathways in peripheral tissues, potentially suppress organismal aging [3]. Phenylalanine is the precursor of tyrosine; specifically, tyrosine is formed through the conversion of phenylalanine mediated by phenylalanine hydroxylase (PAH). Therefore, we also examined the role of phenylalanine. Elevated circulating phenylalanine has been associated with telomere loss [7], inflammatory disease [8], and type 2 diabetes [9]. Experimental evidence shows that phenylalanine can undergo oxidation to form toxic metabolite meta-tyrosine (m-tyrosine), which has been shown to shorten *C. elegans* lifespan [10, 11]. However, the role of phenylalanine and tyrosine in humans has been rarely examined.

Interestingly, lifespan differs by sex. In most regions worldwide, men have a consistently shorter life expectancy compared with women [12], and the disparity may have widened after the COVID-19 pandemic [13]. With US life expectancy declining from 78.8 years in 2019 to 77.0 in 2020 and 76.1 in 2021, the lifespan difference between men and women expanded to 5.8 years, marking the widest gap since 1996 [13]. Notably, tyrosine also differs by sex, with lower levels in young women than in young men [14]. Whether tyrosine explains or partly explains the sex difference in lifespan has not been clarified. In this study, we assessed the associations of tyrosine and its precursor phenylalanine with lifespan in overall people and in men and women separately, using UK Biobank, a large cohort in UK. Since conventional observational designs are inherently susceptible to residual confounding arising from variables such as socioeconomic factors and health status, we also used Mendelian randomization (MR) (Supplementary Figure 1). Using genetic variants as instruments, which are less affected by socioeconomic positions [15], MR has the potential to mitigate confounding. Here we employed MR to assess the role of tyrosine and phenylalanine in lifespan overall and sex-specifically.

## RESULTS

In the cohort study, 272,475 participants with death status information, measurement of amino acids, and information on confounders were included in the analysis. Among these, 125,359 were men. Of these 272,475 participants, 23,964 deaths were identified from death records, including 14,230 in men and 9,734 in women. After adjustment for multiple confounders (details shown in Methods), plasma phenylalanine was linked to elevated all-cause mortality overall (Hazard ratio (HR) 1.04 per SD increase in phenylalanine, 95% confidence interval (CI) 1.03–1.05), in men (HR 1.04, 95% CI 1.02–1.05) and in women (HR 1.04, 95% CI 1.02–1.07). The findings were similar for both men and women. Plasma tyrosine was associated with a higher risk of all-cause mortality overall and in men (HR 1.03, 95% CI 1.01–1.05), but not in women (HR 1.00, 95% CI 0.98–1.03) (Table 1), although the difference in the associations in men and women was not statistically significant (*p* = 0.16).

**Table 1:** The associations of phenylalanine and tyrosine with all-cause mortality in UK Biobank using Cox regression.

**Exposure**	**Sex**	**HR^1^**	**95% CI^2^**	***p***
Phenylalanine	Overall	1.04	1.03, 1.05	1.1 × 10^−9^
	Men	1.04	1.02, 1.05	7.1 × 10^−7^
	Women	1.04	1.02, 1.07	1.2 × 10^−3^
Tyrosine	Overall	1.02	1.00, 1.03	2.1 × 10^−2^
	Men	1.03	1.01, 1.05	5.5 × 10^−3^
	Women	1.00	0.98, 1.03	7.2 × 10^−1^

^1^HR: hazard ratio. In overall analysis we adjusted for age, body mass index (BMI), Townsend Deprivation Index, smoking status, alcohol consumption, physical activity, ethnicity, and education (in years). For combined-sex analyses, we additionally adjusted for sex. ^2^CI: confidence interval.

The associations of phenylalanine and tyrosine with lifespan, both overall and stratified by sex, remained after excluding deaths from accidents (Supplementary Table 1). The Pearson correlation coefficient between phenylalanine and tyrosine was 0.52 (*p* < 0.01). A greater tyrosine-to-phenylalanine ratio was linked to a lower overall risk of all-cause mortality in overall people (HR 0.98, 95% CI 0.97–1.00) and also in women (HR 0.96, 95% CI 0.94–0.99), whereas no association was observed in men (HR 1.00, 95% CI 0.98–1.02). Restricted cubic spline analysis suggested non-linearity, with the turning point at the standardized concentration of around 0 for both amino acids (*p*-value <0.05, Supplementary Figures 2, 3 and Supplementary Table 2). In disease-specific mortality, we found positive associations of phenylalanine with both cardiovascular disease (CVD) mortality (HR 1.03, 95% CI 1.00–1.06) and cancer mortality (HR 1.04, 95% CI 1.02–1.05), whereas tyrosine was not associated with either outcome (Supplementary Table 3). These observations imply that phenylalanine could participate in pathways relevant to cardiovascular health and carcinogenesis.

**Figure 1:**
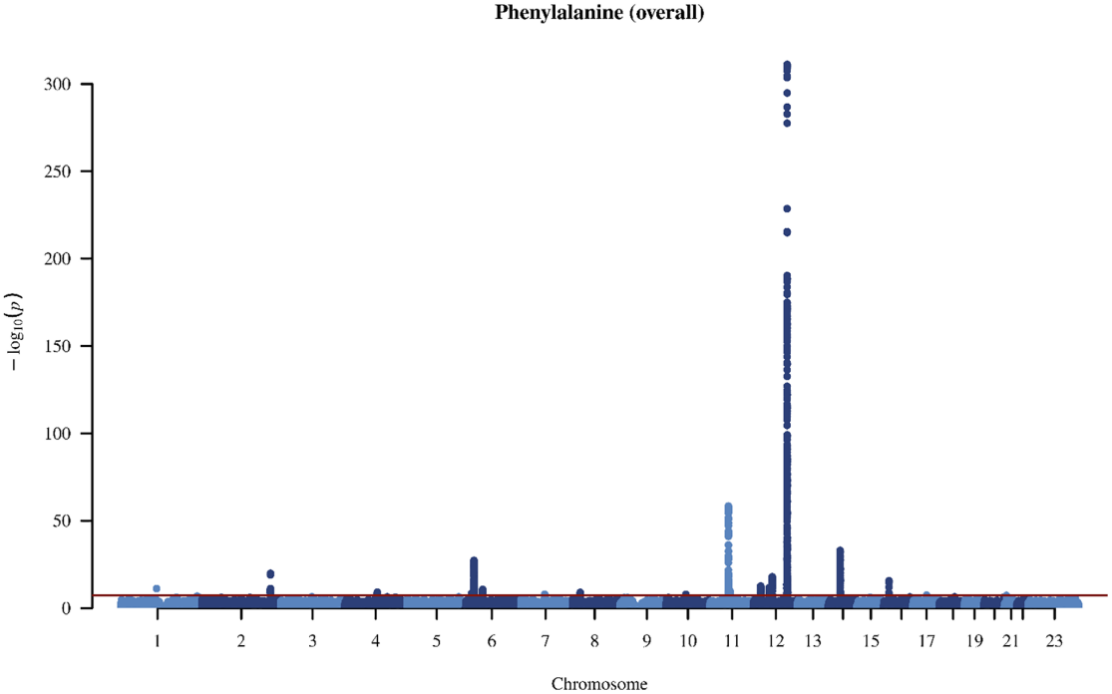
Manhattan plot on the genome-wide association study of phenylalanine in overall people.

**Figure 2:**
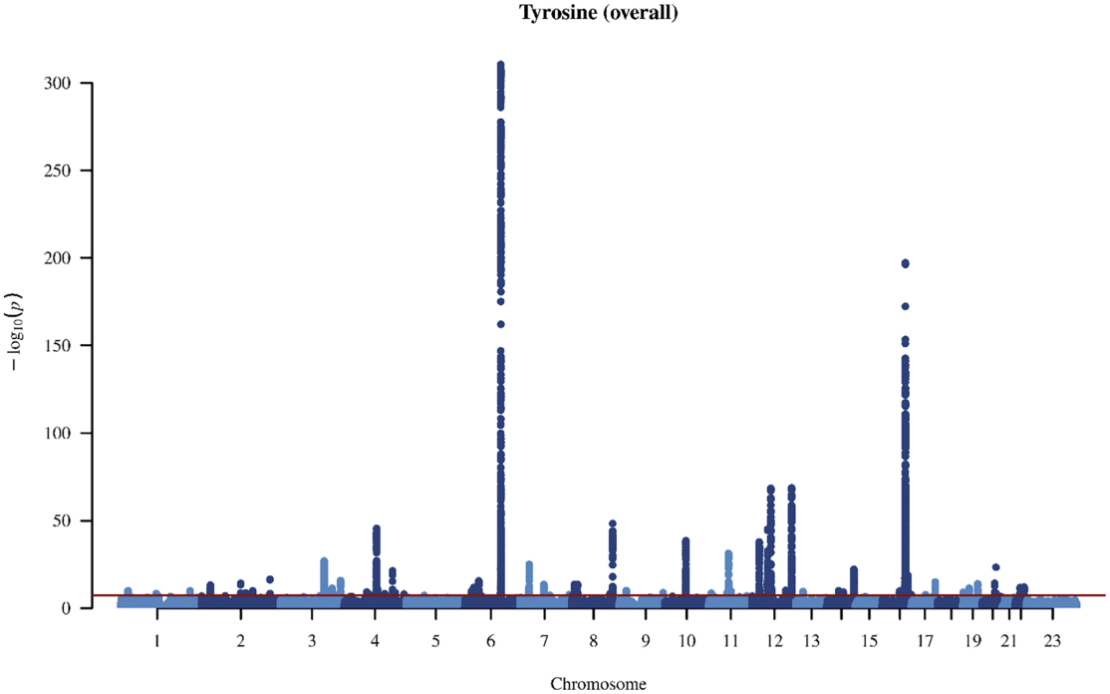
Manhattan plot on the genome-wide association study of tyrosine in overall people.

**Figure 3:**
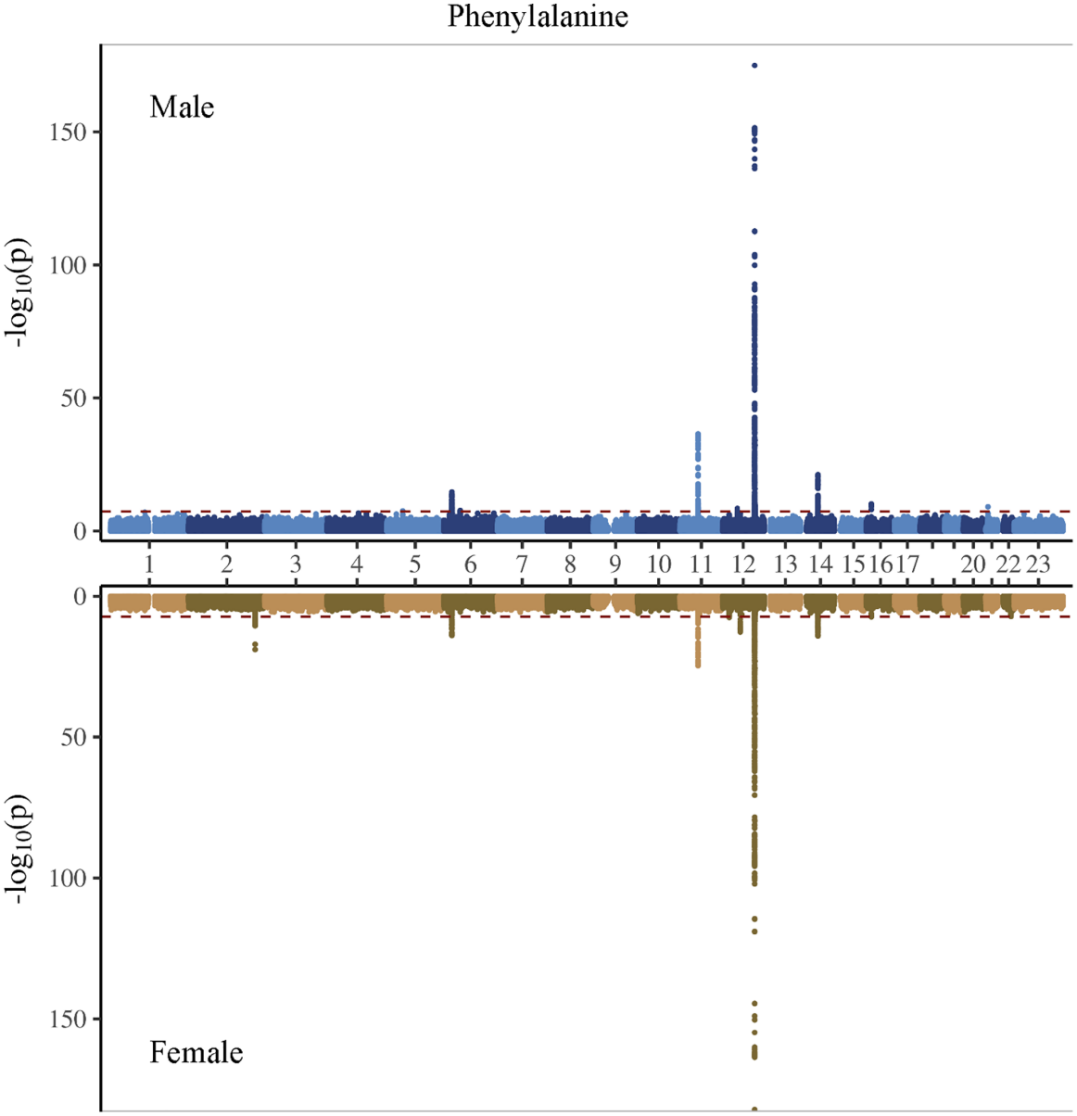
Manhattan plot on the genome-wide association study of phenylalanine in men and women.

**Figure 4:**
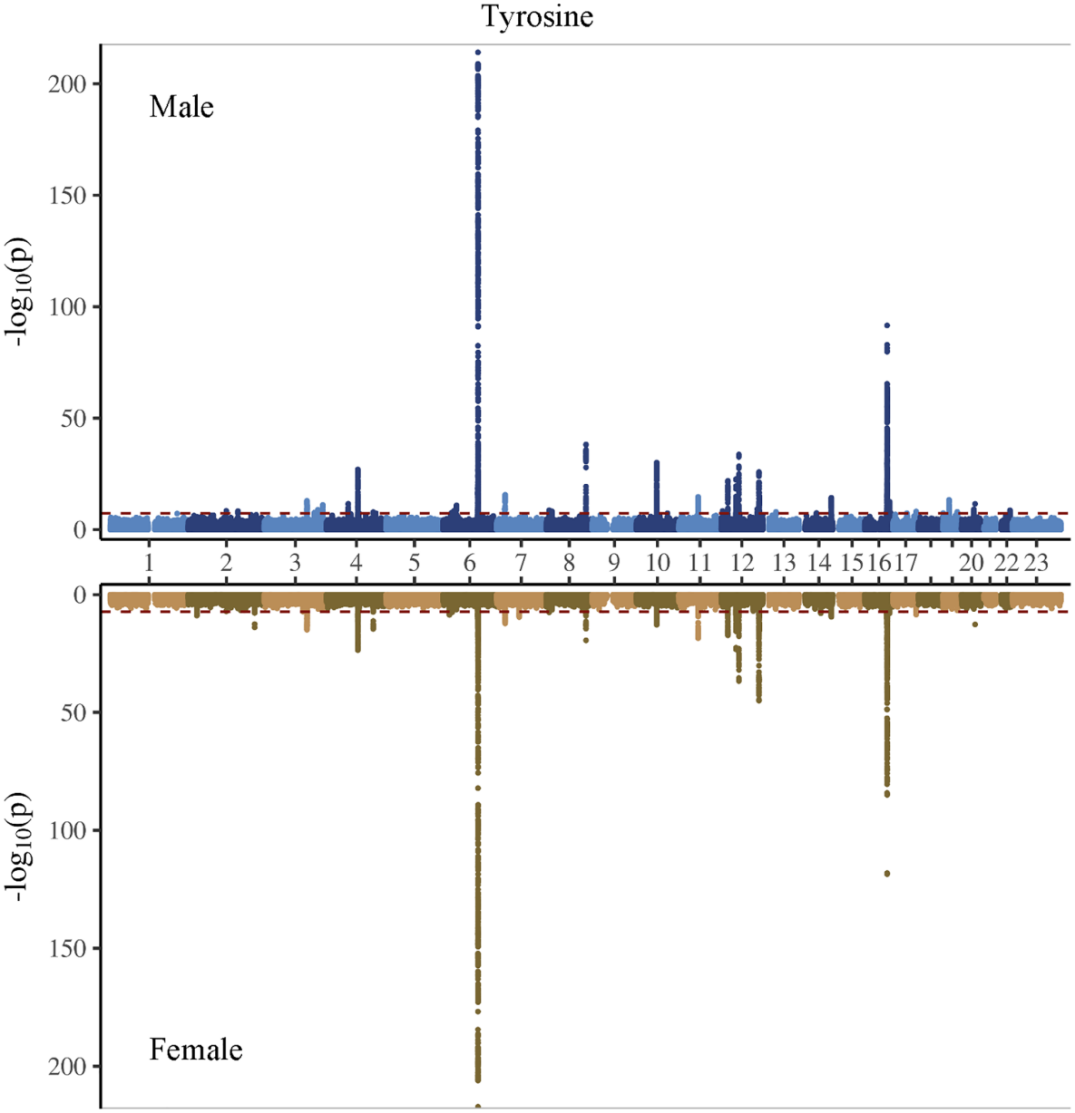
Manhattan plot on the genome-wide association study of tyrosine in men and women.

In the genome-wide association study (GWAS) of two amino acids, the heritability for phenylalanine and tyrosine was 0.04 and 0.09, respectively (Supplementary Table 4). The LDSC intercepts and attenuation ratio indicated no genomic inflation of test statistics due to confounding factors (Supplementary Table 4). The Manhattan plots were shown in Figures 1–4, Quantile-Quantile plots were presented in Supplementary Figure 4. In the overall analysis, we identified 2,422 genetic variants with genome-wide significance for phenylalanine, and 11,379 for tyrosine. In sex-specific GWAS, we identified 1,099 genetic variants for phenylalanine in men and 946 in women, while for tyrosine, 5,297 variants reached genome-wide significance in men and 4,840 in women.

After removing correlated genetic variants, we used 21 and 74 single nucleotide polymorphisms (SNP) as genetic instruments for phenylalanine and tyrosine, respectively in the overall analysis (Supplementary Tables 5 and 6). We used 12 SNPs in men and 10 SNPs in women for phenylalanine, and 45 SNPs in men and 29 SNPs in women for tyrosine (Supplementary Tables 7 and 8). The SNPs associated with phenylalanine and tyrosine are located within genes critical for amino acid metabolism, transport, and regulation. For phenylalanine, essential genes include *PAH*, which catalyzes phenylalanine’s conversion to tyrosine; members of the solute carrier (*SLC*) transporter family (*SLC17A1*, *SLC38A4*, and *SLC43A1*), which facilitate cellular uptake and distribution of amino acids; and carbamoyl-phosphate synthase 1 (*CPS1*), an essential enzyme in the urea cycle linking nitrogen metabolism with amino acid catabolism. Additionally, genes in the glutathione S-transferase (*GST*) family, including *GSTM1* and *GSTA2*, encode enzymes central to detoxification pathways by conjugating amino acid-derived metabolites. For tyrosine, SNPs further involve the previously highlighted genes *PAH*, *CPS1* and *GSTM1*, alongside *HPD* encoding 4-hydroxyphenylpyruvate dioxygenase, which participates in tyrosine breakdown through homogentisate formation.

**Figure 5:**
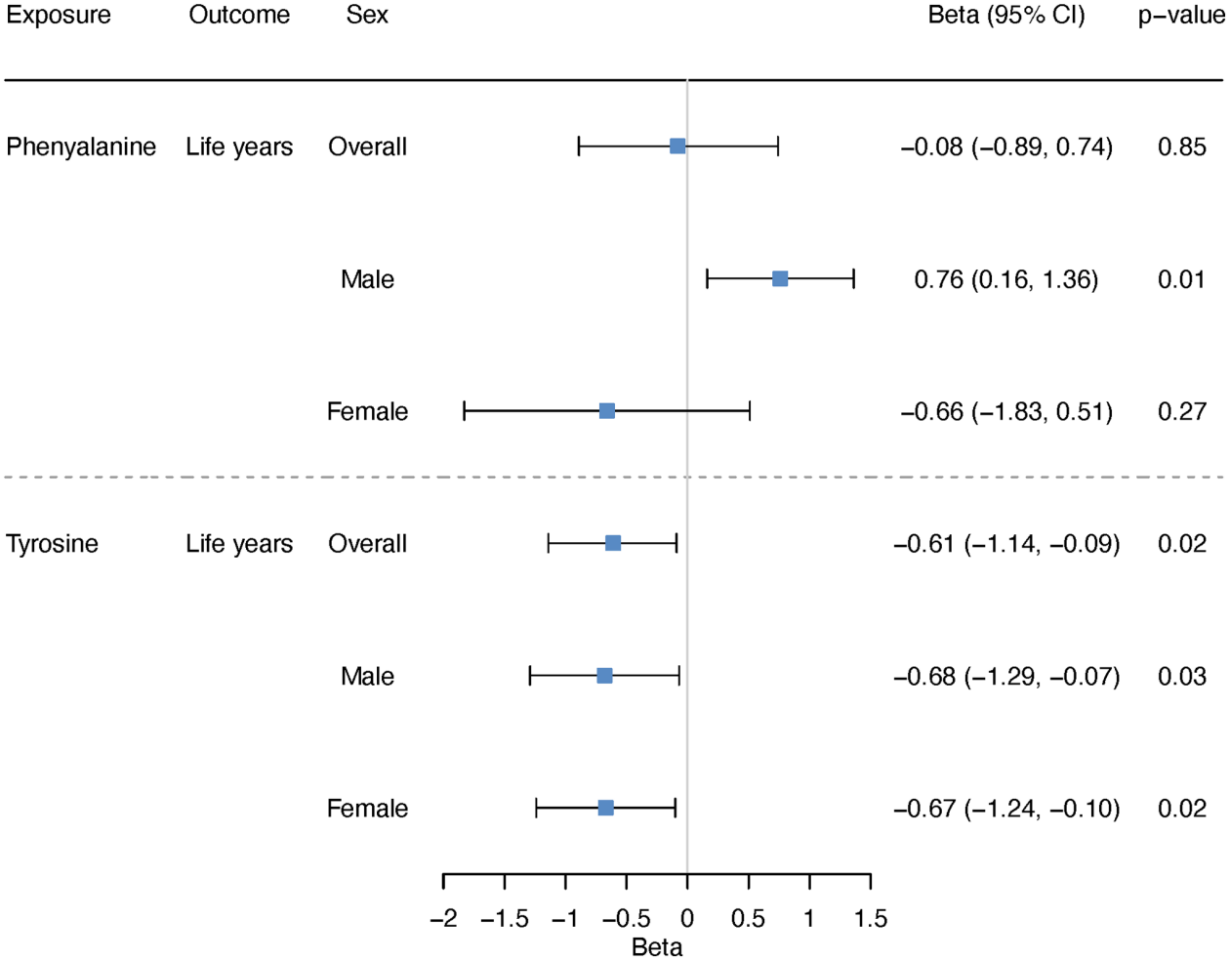
**Overall and sex-specific associations of phenylalanine and tyrosine with lifespan using inverse variance weighting.** We presented increased/decreased life years for ease of understanding; these estimates were calculated based on the log hazard ratios reported by the lifespan GWAS (detailed described in “Methods-Genetic associations with lifespan”).

**Figure 6:**
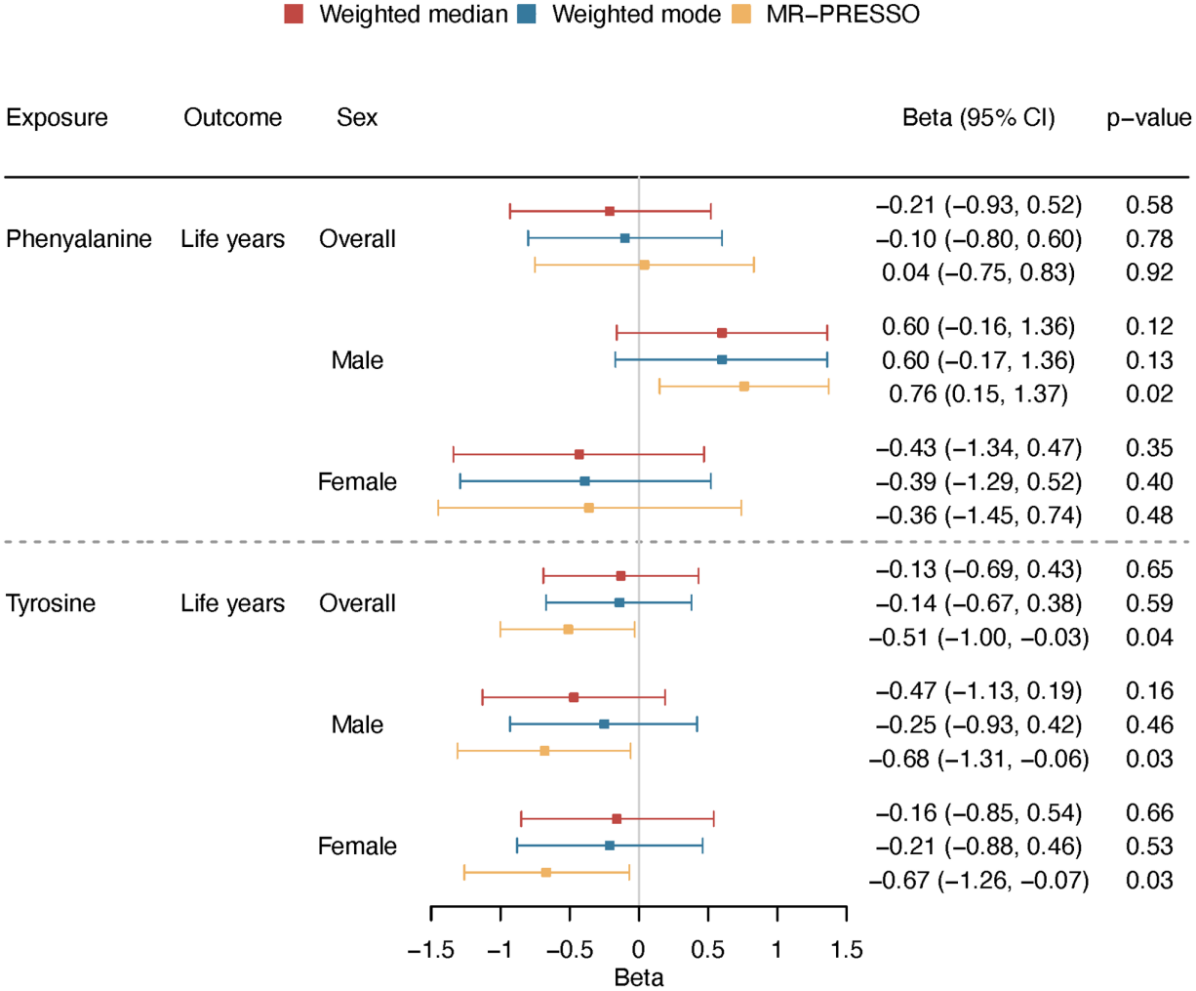
**Overall and sex-specific associations of phenylalanine and tyrosine with lifespan using different analytic methods (weighted median, weighted mode and MR-PRESSO).** We presented increased/decreased life years for ease of understanding; these estimates were calculated based on the log hazard ratios reported by the lifespan GWAS (detailed described in “Methods-Genetic associations with lifespan”).

Using two-sample MR, we estimated the effect on lifespan, i.e., years of life. Genetically predicted higher phenylalanine was related to longer lifespan in men but not related to lifespan in overall analysis or in women (Figure 5). The association in men showed consistent directions of associations applying various analytic methods (Figure 6). Genetically mimicked higher tyrosine levels were linked to a shorter lifespan in the overall population and in both sexes using inverse variance weighting (IVW) (Figure 5). The associations were also shown when we used Mendelian randomization pleiotropy residual sum and outlier (MR-PRESSO) (Figure 6), after excluding outliers (Supplementary Table 9 and Figure 6). The associations in weighted median and weighted mode showed aligned directions of association, but the CI included the null (Figure 6). The associations persisted after the exclusion of SNPs with potential pleiotropy (Supplementary Table 10). Scatter plot and leave-one-out plot provided no indication that the relationships were affected by any individual SNP (Supplementary Figures 5 and 6). Sensitivity analysis using genetic instruments from another GWAS in overall people without UK Biobank participants showed consistent directions of associations (Supplementary Table 11). Genetically predicted phenylalanine was linked to longer lifespan in men, whereas no relationship was observed in the overall people or among women. Genetically predicted tyrosine had an inverse association with lifespan overall using MR-PRESSO and had the direction of inverse association in men and women especially using MR-PRESSO (Supplementary Table 11). Power calculation showed that at 80% statistical power, we can identify an effect size of ~1.6 life years for phenylalanine and 1.0 life years for tyrosine (Supplementary Table 12).

In multivariable MR study including both amino acids, we found that after controlling for tyrosine, phenylalanine was not related to lifespan. Interestingly, after controlling for phenylalanine, tyrosine was associated with shorter lifespan in men, while no clear relationship in women (Table 2). The positive association was shown in both IVW and MR Egger in men, but not shown in MR Egger in women (Table 2). MR Egger results provided no indication of directional pleiotropy (intercept *p* > 0.05).

**Table 2:** The sex-specific associations of genetically predicted phenylalanine and tyrosine with lifespan using multivariable MR, including inverse variance weighting (IVW) and MR-Egger.

**Sex**	**Methods**	**Exposure**	**Life years**	**95% CI**	***p***	**MR-Egger intercept *p***
Men	IVW	phenylalanine	0.75	−0.07, 1.58	0.08	0.61
		tyrosine	−0.80	−1.40, −0.23	0.006	
	MR-Egger	phenylalanine	0.55	−0.59, 1.69	0.35	
		tyrosine	−0.91	−1.60, −0.21	0.01	
Women	IVW	phenylalanine	−0.72	−1.47, 0.05	0.07	0.15
		tyrosine	−0.59	−1.11, −0.05	0.03	
	MR-Egger	phenylalanine	−0.18	−1.24, 0.85	0.73	
		tyrosine	−0.36	−0.96, 0.23	0.24	

## DISCUSSION

Our novel finding contributes to the scarce epidemiological evidence regarding the role of tyrosine and phenylalanine in lifespan. Our study showed that tyrosine was associated with shorter lifespan in observational and MR studies. The association was independent of phenylalanine, which remained in multivariable MR after controlling for phenylalanine. The role of tyrosine may be sex-specific, with a clearer effect in men than in women. Phenylalanine was not related to lifespan after controlling for tyrosine.

Based on our results, targeting tyrosine may be a potential strategy for improving lifespan. Partly consistent with our findings, animal experiment suggests that restricting dietary protein in rats extends lifespan while lowering tyrosine concentrations in liver and muscle [16]. The biological processes linking tyrosine to lifespan have not been thoroughly determined. Tyrosine was associated with insulin resistance [17]. According to evolutionary biology, more investment in growth and reproduction often comes at the expense of lifespan [18], while insulin acts as one of key regulators of growth and reproduction [19, 20]. Consistently, insulin resistance has been shown to be related to multiple diseases and decreased lifespan [21]. Insulin resistance may also have sex-specific effects [22, 23]. Restricting caloric intake, known to reduce the risk of insulin resistance [24], also prolongs lifespan in a sex-specific way [25]. Insulin resistance may interact with sex hormones, and testosterone has been shown to be related to survival, with a more obvious effect in men than in women [26]. Meanwhile, tyrosine acts as a precursor for neurotransmitters such as dopamine, norepinephrine, and epinephrine [4], which are crucial for regulating mood, cognition, and stress responses [5] and potentially influencing lifespan [6]. Interestingly, these neurotransmitters are regulated by sex hormones [27, 28], which provides another explanation for the sex-specific associations.

Our study, for the first time, evaluated the role of tyrosine and phenylalanine in lifespan using both conventional observational study and MR. In this novel study, we also examined the sex difference in the associations, and suggested potential sex disparity in the role of tyrosine. Despite of the novelty, our study bears some limitations. First, traditional observational study, including our study, is inevitably susceptible to residual confounding. Some confounders, such as socioeconomic position, is difficult to be accurately measured [29]. In contrast, MR study can minimize confounding by leveraging genetic variants that are randomly assigned at conception [30]. This may partly explain the inconsistent associations for phenylalanine in observational study and MR study. Second, MR required stringent assumptions: relevance, independence, and exclusion-restriction. Accordingly, we selected genetic instruments with strong associations with these amino acids. In addition, we tested the associations of these genetic instruments with potential confounders. Considering that phenylalanine and tyrosine have shared SNPs, including rs140584594, rs10750864 and rs1043011, we also conducted multivariable MR to examine the independent role of phenylalanine and tyrosine. Third, genetic associations with the amino acids and with lifespan are both from UK Biobank, the sample overlap could introduce bias into the MR estimates. However, our sensitivity analysis leveraging genetic variants derived from GWAS conducted in combined-sex populations outside the UK Biobank showed consistent directions of associations. It would be ideal to replicate using sex-specific GWAS not conducted in UK Biobank, but such GWAS was not available yet. Moreover, a recent study suggested that MR analyses using overlapped samples in large cohorts like UK Biobank can still provide valid estimates [31]. Fourth, the study may lack adequate power to identify sex difference in the role of tyrosine, which may explain the marginal significance in the testing for sex disparity on the associations of tyrosine. Given the consistent trends observed in both observational and multivariable MR studies, it is more plausible that the marginal significance reflects limited statistical power, not an actual lack of effect. This is further supported by power calculations. Therefore, replicating the study in larger cohorts would be worthwhile. Fifth, MR study assessed the role of endogenous exposures, which is different from nutrient supplementation. While blood levels of amino acids respond to nutrient supplementation or diet rich in these amino acids [32–34], our findings on circulating tyrosine or phenylalanine may not directly reflect the role of dietary consumption of these amino acids. Sixth, these amino acids were only measured at a single time point. Future investigations with repeated measures would be valuable to further elucidate how circulating phenylalanine and tyrosine levels fluctuate over time and to clarify their influence on mortality outcomes. Seventh, our findings need to be interpreted with caution. Given the potential non-linearity, the positive associations with mortality are more applicable to people with higher levels of phenylalanine or tyrosine. Replicating these results in populations with different levels of amino acids would be worthwhile. Finally, MR study examined the lifelong effect of phenylalanine and tyrosine, which is not comparable to randomized controlled trials assessing short-term effects of supplementation.

From the perspective of etiology, our study suggests that tyrosine is involved in longevity. More mechanistic studies will be worthwhile to assess the possible pathways. The circulating level of tyrosine is modifiable. In terms of public health interventions, our findings indicate that nutrients or diets, such as protein-restriction diet, which lower tyrosine will be helpful for prolonging lifespan. Tyrosine is also a popular nutrient supplement, promoted as a neurotransmitter support for a positive mood and mental alertness. Our study is not directly related to tyrosine supplement, but given tyrosine supplement may increase blood tyrosine, our study did not support the benefit of long-term use of tyrosine on lifespan.

## METHODS

### Study design

To understand the role of phenylalanine and tyrosine in longevity, we used conventional observational study to examine their relationships with all-cause mortality in the UK Biobank. To minimize confounding, we applied univariable MR to assess the associations of genetically predicted phenylalanine and tyrosine with parental attained age. Given that phenylalanine and tyrosine are correlated, we further performed multivariable MR to examine their independent effects. To assess the sex-specific roles, we conducted sex-stratified analyses in both observational and MR studies. The study design was shown in the flow diagram in Supplementary Figure 1.

### Cohort study

UK Biobank is a large-scale cohort study, with a current median follow up of 11.1 years [35]. Between 2006 and 2010, it enrolled 502,713 individuals aged 40–69 years, with a mean age of 56.5 years in England, Scotland and Wales. Among all participants, 45.6% are men and 94% were identified as of European ancestry by self-report. Utilizing data from UK Biobank, we studied the associations of baseline plasma levels of phenylalanine and tyrosine with all-cause mortality using Cox regression, controlling for age, sex (in the overall analysis but not in sex-specific analysis), Townsend index, smoking habits, alcohol intake, physical activity, self-reported ethnicity (white, black, Asian, and other), education (years) and body mass index (BMI). Deaths were identified by death records. We also conducted sensitivity analysis excluding deaths from accidents (V00–Y99). To assess the potential nonlinear associations, we used restricted cubic splines [36]. We also examined the correlation between phenylalanine and tyrosine and the association of tyrosine-to-phenylalanine ratio with all-cause mortality. We set the censoring date to 19 Dec 2022, which is the latest date of death in the records. In addition to examining all-cause mortality, we also investigated disease-specific mortality outcomes based on the International Classification of Diseases (ICD-10) codes. Specifically, we assessed the associations with CVD mortality (I00–I99) and cancer mortality (C00–D48), the top two leading contributors to mortality in UK Biobank. Plasma levels of phenylalanine and tyrosine were quantified in absolute concentrations (mmol/L), measured in a high-throughput NMR-based metabolic biomarker profiling platform (Nightingale Health Ltd.). Procedures for sample preparation, spectrometer calibration, and quality-control protocols are detailed in previous publications [35, 37, 38]. All measures were standardized before analyses.

### MR study

#### Overall and sex-specific GWAS of phenylalanine and tyrosine

We conducted a GWAS of the plasma levels of phenylalanine and tyrosine in the UK Biobank utilizing fastGWA tool (GCTA toolbox, version 1.94.1) [39]. In the mixed linear model association analyses, we utilized a sparse genetic relationship matrix with a cutoff value of 0.05, which was computed from linkage disequilibrium (LD)-pruned HapMap 3 SNP set. The LD-pruning parameters set in PLINK included a window of 1,000 variants, step size of 100, r^2^ threshold of 0.9, and minor allele frequency exceeding 0.01 [40]. For our genome-wide association analyses, we excluded SNPs with an imputation score below 0.3, minor allele frequency under 0.1%, missing genotype rates exceeding 5% per individual, missing genotype rates over 5% per genetic variant, or *p*-value of Hardy-Weinberg equilibrium less than 1 × 10^−8^. In the non-pseudoautosomal X chromosome region, males were coded as 0 or 2 copies of the effect allele. Participants of European ancestry were characterized in the Pan-ancestry genetic analysis of the UK Biobank (Pan-UK Biobank) [41]. Additionally, participants were not included in the analysis if they had withdrawn consent, displayed discrepancies between self-reported and genetic sex, exhibited sex chromosome aneuploidy, were identified as heterogeneity outliers or missing genotype rate. After quality control, we performed both combined and sex-stratified GWAS of phenylalanine and tyrosine. In the sex-specific GWAS, age and 10 genetic principal components supplied by the Pan-UK Biobank were included as covariates, while sex was added as an additional covariate in the combined-sex GWAS. We applied the rank-based inverse normal transformation to phenylalanine and tyrosine measurements to enable interpretation per one standard deviation (SD) change [42]. We computed the SNP-based heritability and checked for inflation by LD score regression [43].

#### Genetic instruments for phenylalanine and tyrosine

Genetic proxies for circulating phenylalanine and tyrosine were obtained based on the GWAS we conducted in the UK Biobank. Specifically, we selected SNPs linked to circulating phenylalanine or tyrosine reaching genome-wide significance (5 × 10^−8^) and meeting an LD cutoff of r^2^ < 0.001. The instruments for overall analysis were based on GWAS in the overall sample, whilst the genetic instruments for sex-specific analyses were derived from the corresponding sex-specific GWAS. To ensure the validity of the genetic variants, we verified that the F-statistic exceeded 10 [44], with the F-statistic derived from a commonly used formula [45]. The selected genetic instruments were presented in Supplementary Tables 5–8. To understand the potential pleiotropy, we examined whether these selected SNPs were associated with potential confounders for the association between phenylalanine or tyrosine and all-cause mortality, such as Townsend index, education, smoking status, alcohol consumption and physical activity in the UK Biobank. SNPs showing genome-wide significant associations with any of these factors were excluded in sensitivity analysis, as shown in Supplementary Tables 13 and 14.

#### Genetic associations with lifespan

Lifespan was used as the outcome. We retrieved genetic associations for parental attained age (age at death or current age) from a large-scale GWAS involving 389,166 UK Biobank participants of European ancestry [46]. Utilizing parental lifespan is a common way in GWAS of longevity [46], as longevity is heritable [47], so parental lifespan can provide a proxy measure for offsprings’ lifespan, and it can be used even when participants are still alive. The combined parental attained age was calculated by adding the z-standardized maternal and paternal attained ages [46]. The GWAS controlled for age, sex, and the first five principal components [46]. Genetic associations for paternal and maternal attained age were obtained from sex-specific GWAS of parental longevity in participants of European descent from the UK Biobank (fathers: *n* = 415,311; mothers: *n* = 412,937) [46]. Employing sex-stratified Cox proportional hazards model, the GWAS estimated the effect of offspring genetic variant on parental survival, adjusting for age as well as 10 principal components of ancestry. To enhance interpretability, GWAS summary statistics (log hazard ratio) were transformed into years of life through sign inversion and multiplication by 10 [46, 48]. Considering the effect sizes derived from offspring genetic data represent half the true parental variant effect, we doubled the log hazard ratios in the overall analyses [46], and multiplied by 2.2869 for fathers and 2.5863 for mothers, respectively, in the sex-specific analyses [46, 49], as previously described [50].

### Statistical analysis

In the univariable MR, SNP-specific estimates were derived from Wald ratios, which were calculated as the genetic association with parental attained age divided by the association with phenylalanine or tyrosine. These ratios were integrated via IVW with multiplicative random effects [51]. For the sex-stratified analysis, we utilized the genetic associations from sex-specific GWAS of lifespan and phenylalanine or tyrosine. The MR estimates were presented as life years per SD increase in phenylalanine or tyrosine. Multiple comparisons were accounted for using a false discovery rate (FDR) threshold of less than 0.05. Associations showing nominal significance (*p* < 0.05) that failed multiple testing correction were defined as suggestive associations. To assess whether the sex difference has statistical significance, we performed the heterogeneity test with the “meta” package in R.

To address possible pleiotropy, as previously [52–55], we applied multiple analytic approaches robust to pleiotropy, such as the weighted median, weighted mode, MR-PRESSO and MR-Egger methods. The weighted median approach offers a reliable estimate of the causal effect even if as much as half of the information comes from SNPs that invalid instruments [56]. Weighted mode assumes that the largest group of are valid, that is, no larger group of invalid instruments providing the same causal estimate exists [57]. MR-PRESSO detects and removes outlier SNP(s) that disproportionately influenced associations [58], and gives the corrected estimates after the removal of the outliers. MR-Egger can determine if genetic variants exhibit directional pleiotropy, that is, whether their pleiotropic effects on the outcome deviate from zero on average, as indicated by a non-zero intercept, and it also provides a corrected estimate [59]. However, this approach usually gives wider confidence intervals compared to other methods [60]. Considering the overlapping in samples of GWAS for exposure and outcomes, we additionally performed a sensitivity analysis utilizing SNPs for phenylalanine and tyrosine derived from a GWAS which does not include participants from UK Biobank [61], but only overall GWAS is available.

#### Multivariable MR

In addition to univariable MR analyses, we conducted multivariable MR, which leverages pleiotropic SNPs associated with more than one exposure, to assess the causal effects of individual exposure adjusting for other exposure(s) [62]. We included genetic instruments for both phenylalanine and tyrosine, to examine the independent effect of phenylalanine and tyrosine. The genetic instruments for each amino acid were as used in univariable MR. After integrating the SNPs for both amino acids, we removed overlapping and correlated (r^2^ > 0.05) SNPs, and the remaining genetic variants were utilized for the multivariable MR analysis. We employed multivariable MR-Egger analysis to detect directional pleiotropy, and when it was identified, we adopted the multivariable MR-Egger estimates as the main analysis results [63].

#### Power calculation

In power calculation, the required sample size for MR studies is roughly the conventional observational study sample size divided by the proportion of variance in the exposure explained by the genetic instruments [64]. Variance explained by individual SNP was computed via beta^2^ × 2 × (EAF) × (1-EAF), with beta as the effect allele’s standardized beta coefficient, EAF as its frequency [65]. For lifespan, we first calculated the detected effect size at current sample size (i.e., log odds ratio) based on case/non-case ratio, total sample size, and the variance explained by SNPs [66], and then converted to life years using the same way as we did in the statistical analysis.

All statistical analyses were performed using the R packages “TwoSampleMR”, “MendelianRandomization”, “MRPRESSO” and “meta” (R version 4.0.1, R Foundation for Statistical Computing, Vienna, Austria).

### Availability of data and materials

The dataset supporting the conclusions of this article is available upon request and approval by the UK Biobank (https://www.ukbiobank.ac.uk/enable-your-research/apply-for-access).

## Supplementary Materials

Supplementary Figures

Supplementary Tables
