# Supplementary material for: The role of phenylalanine and tyrosine in longevity: a cohort and Mendelian randomization study
Source: Aging (Albany NY). 2025 Oct 3;17(10):2500–33. doi: 10.18632/aging.206326 (PMC12606968; doi:10.18632/aging.206326)
Supplement: Supplementary Figures [file aging-17-10-206326-s001.pdf]

SUPPLEMENTARY FIGURES

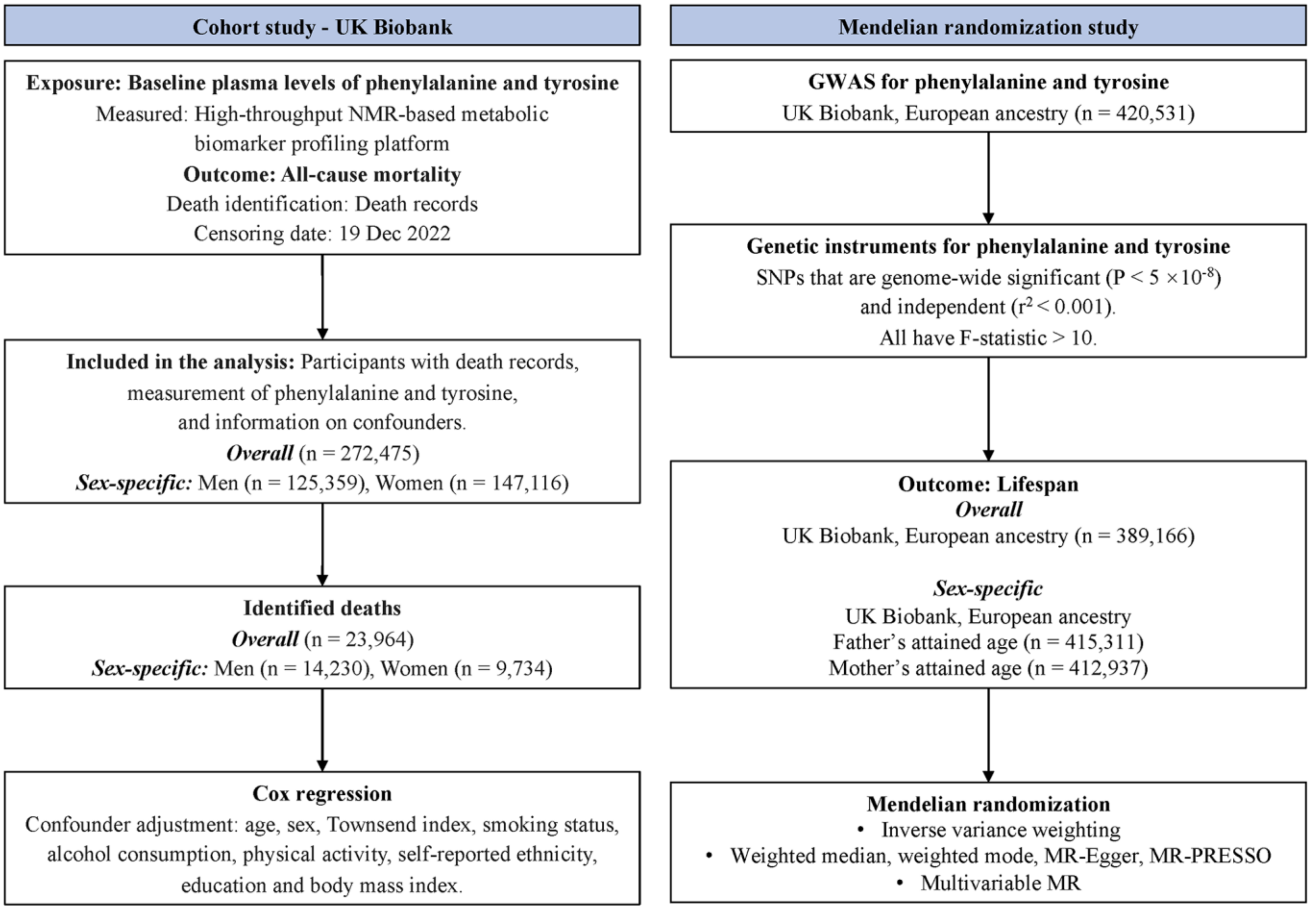

Supplementary Figure 1. Flow chart of study design.

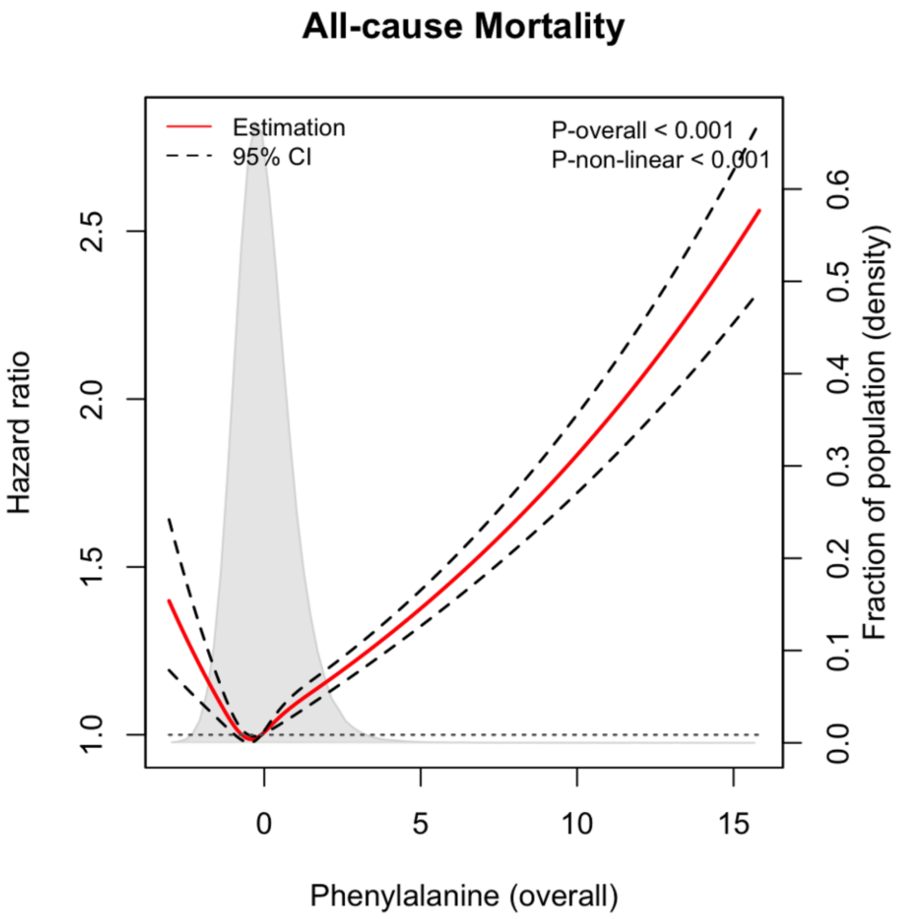

Supplementary Figure 2. Association of phenylalanine with all-cause mortality using restricted cubic splines.

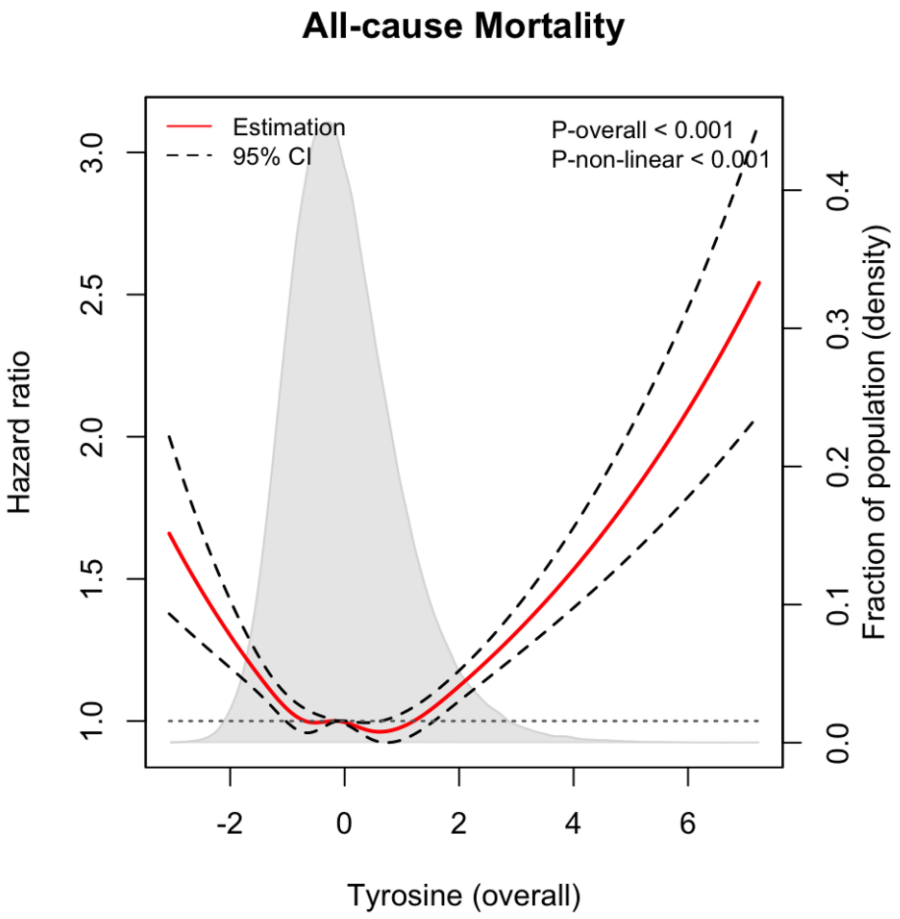

Supplementary Figure 3. Association of tyrosine with all-cause mortality using restricted cubic splines.

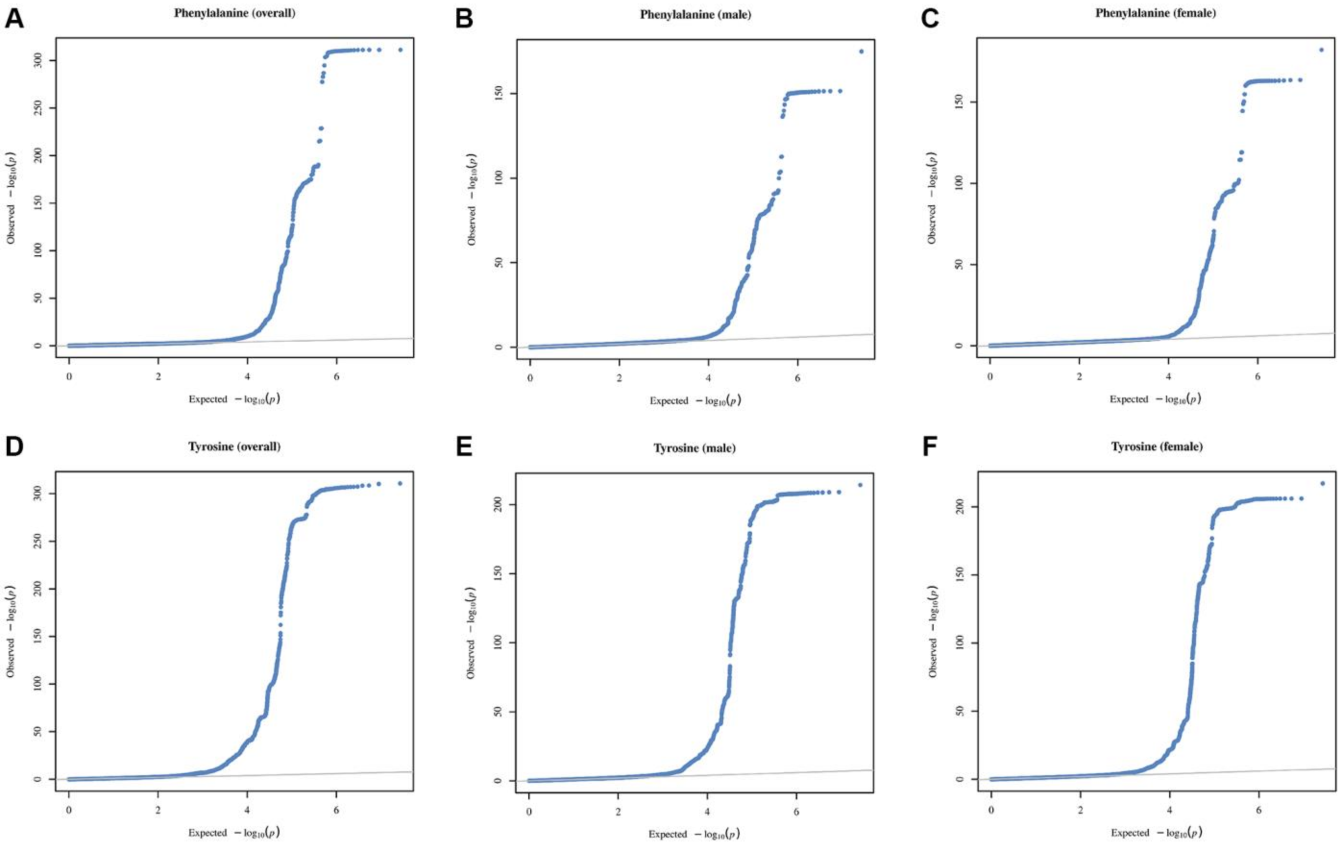

Supplementary Figure 4. Q-Q plot on the genome-wide association study of phenylalanine and tyrosine in overall people, men and women. (**A**) Q-Q plot for phenylalanine in overall people; (**B**) Q-Q plot for phenylalanine in men; (**C**) Q-Q plot for phenylalanine in women; (**D**) Q-Q plot for tyrosine in overall people; (**E**) Q-Q plot for tyrosine in men; (**F**) Q-Q plot for tyrosine in women.

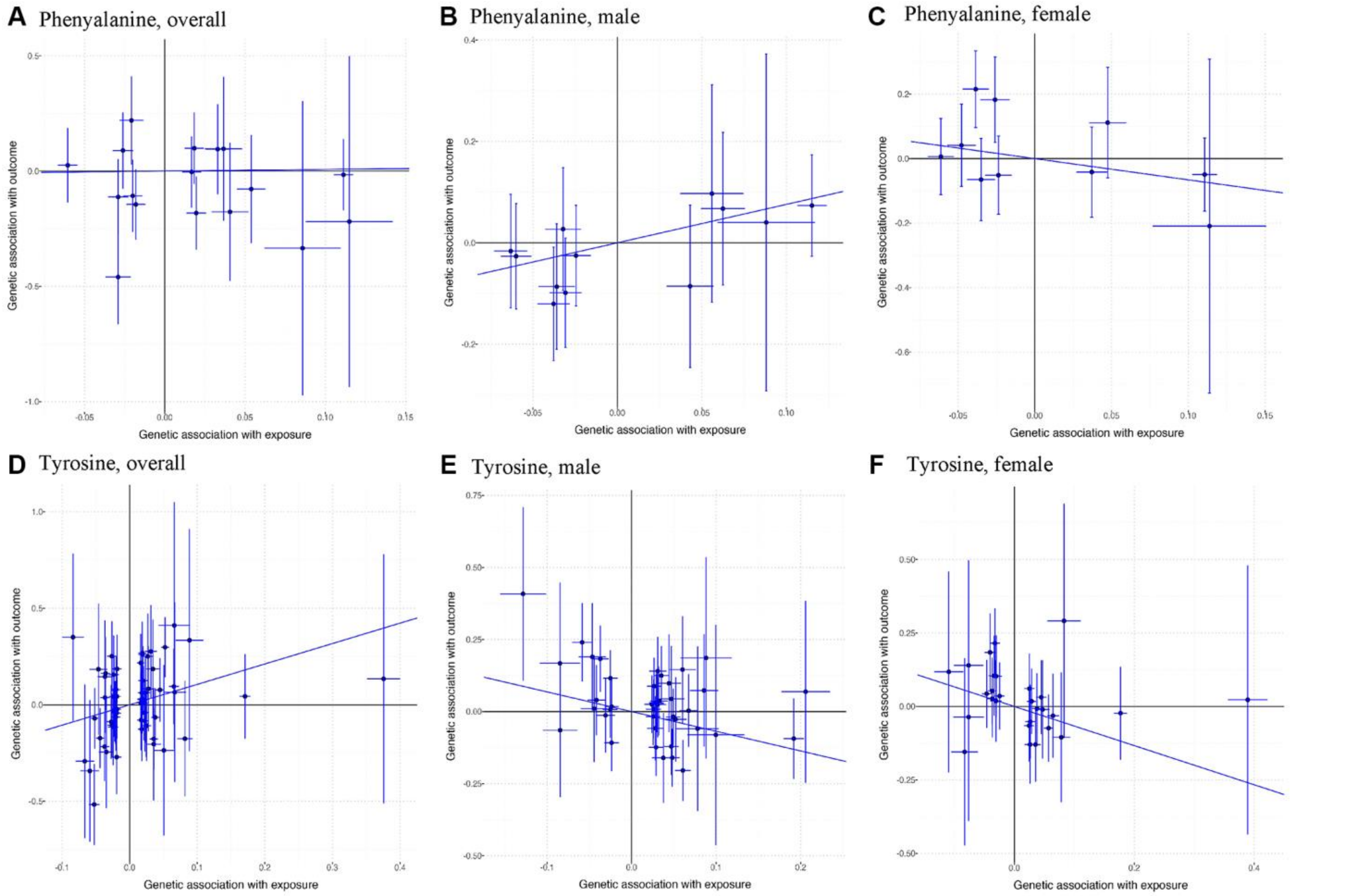

Supplementary Figure 5. Scatter plots on the associations of each SNP with amino acids and with lifespan in overall people, men and women. (**A**) Scatter plot for phenylalanine in overall people; (**B**) Scatter plot for phenylalanine in men; (**C**) Scatter plot for phenylalanine in women; (**D**) Scatter plot for tyrosine in overall people; (**E**) Scatter plot for tyrosine in men; (**F**) Scatter plot for tyrosine in women.

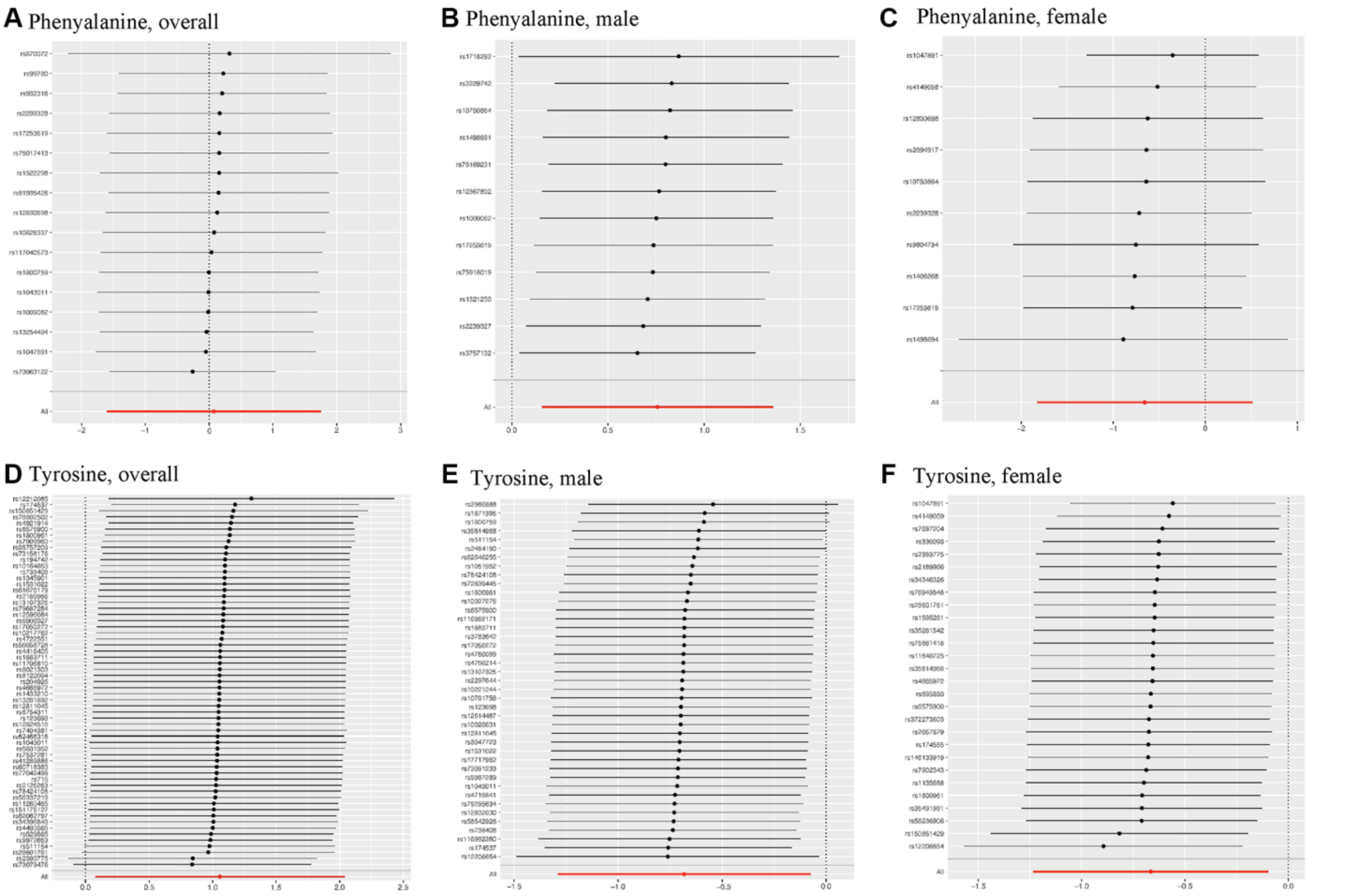

Supplementary Figure 6. Leave-one-out analysis on the associations of phenylalanine and tyrosine with lifespan. (**A**) Leave-one-out analysis for phenylalanine in overall people; (**B**) leave-one-out analysis for phenylalanine in men; (**C**) leave-one-out analysis for phenylalanine in women; (**D**) leave-one-out analysis for tyrosine in overall people; (**E**) leave-one-out analysis for tyrosine in men; (**F**) leave-one-out analysis for tyrosine in women.
