# Supplementary material for: The role of phenylalanine and tyrosine in longevity: a cohort and Mendelian randomization study
Source: Aging (Albany NY). 2025 Oct 3;17(10):2500–33. doi: 10.18632/aging.206326 (PMC12606968; doi:10.18632/aging.206326)
Supplement: Supplementary Tables [file aging-17-10-206326-s002.pdf]

SUPPLEMENTARY TABLES

Supplementary Table 1. The associations of phenylalanine and tyrosine with all-cause mortality excluding deaths from accidents in UK Biobank using Cox regression.

Exposure	Sex	HR[1]	95% CI[2]	*p*
Phenylalanine	Overall	1.04	1.03, 1.05	3.3×10^{-9}
	Men	1.04	1.02, 1.05	3.2×10^{-7}
	Women	1.04	1.02, 1.07	8.7×10^{-4}
Tyrosine	Overall	1.02	1.00, 1.03	1.9×10^{-2}
	Men	1.03	1.01, 1.05	3.4×10^{-3}
	Women	1.00	0.98, 1.03	7.8×10^{-1}

[1]HR: hazard ratio. In overall analysis we adjusted for age, body mass index (BMI), Townsend Deprivation Index, smoking status, alcohol consumption, physical activity, ethnicity, and education (in years). For combined-sex analyses, we additionally adjusted for sex. [2]CI: confidence interval.

Supplementary Table 2. The associations of phenylalanine and tyrosine with all-cause mortality stratified by amino acid level in UK Biobank using Cox regression.

Exposure	Stratified by amino acids levels[1]	HR[2]	95% CI[3]	*p*
Phenylalanine	Lower level	0.95	0.91, 0.99	2.5×10^{-2}
	Higher level	1.05	1.04, 1.06	8.4×10^{-17}
Tyrosine	Lower level	0.89	0.85, 0.93	2.7×10^{-13}
	Higher level	1.10	1.07, 1.13	4.5×10^{-7}

[1]Higher or lower level means above or below standardized concentration of 0 for phenylalanine and tyrosine. [2]HR: hazard ratio. We adjusted for age, sex, BMI, Townsend Deprivation Index, smoking status, alcohol consumption, physical activity, ethnicity, and education (in years). [3]CI: confidence interval.

Supplementary Table 3. The associations of phenylalanine and tyrosine with cardiovascular disease (CVD) and cancer mortality in UK Biobank using Cox regression.

Exposure	Outcome	Sex	HR[1]	95% CI[2]	*p*
Phenylalanine	CVD mortality	Overall	1.03	1.00, 1.06	3.4×10^{-2}
		Men	1.03	0.99, 1.07	1.7×10^{-1}
		Women	1.05	0.99, 1.12	9.3×10^{-2}
Tyrosine	CVD mortality	Overall	1.01	0.98, 1.04	6.3×10^{-1}
		Men	1.01	0.97, 1.05	7.0×10^{-1}
		Women	1.00	0.94, 1.07	9.8×10^{-1}
Phenylalanine	Cancer mortality	Overall	1.04	1.02, 1.05	6.3×10^{-5}
		Men	1.04	1.01, 1.06	2.7×10^{-3}
		Women	1.04	1.01, 1.07	2.2×10^{-2}
Tyrosine	Cancer mortality	Overall	1.02	0.99, 1.04	1.6×10^{-1}
		Men	1.01	0.99, 1.04	3.3×10^{-1}
		Women	1.02	0.99, 1.06	1.9×10^{-1}

[1]HR: hazard ratio. In overall analysis we adjusted for age, BMI, Townsend Deprivation Index, smoking status, alcohol consumption, physical activity, ethnicity, and education (in years). For combined-sex analyses, we additionally adjusted for sex. [2]CI: confidence interval.

Supplementary Table 4. Linkage disequilibrium score regression.

Phenotype	Population	Sex	h^2	h^2_se	lambda	intercept	intercept_se	ratio	ratio_se
Phenylalanine	EUR	Overall	0.036	0.009	1.121	1.028	0.009	0.143	0.043
Phenylalanine	EUR	Female	0.031	0.008	1.068	1.014	0.008	0.145	0.080
Phenylalanine	EUR	Male	0.042	0.010	1.059	1.011	0.009	0.110	0.082
Tyrosine	EUR	Overall	0.087	0.018	1.250	1.062	0.011	0.120	0.022
Tyrosine	EUR	Female	0.080	0.023	1.127	1.040	0.009	0.153	0.035
Tyrosine	EUR	Male	0.114	0.026	1.152	1.035	0.010	0.114	0.032

Supplementary Table 5. Genetic instruments for phenylalanine in the overall analysis.

SNP	effect_allele	other_allele	gene	beta	se	*p*-value	F-statistics
rs1009062	G	T	*GSTA2*	−0.02	0.003	1.76E-11	45.2
rs1043011	T	G	*GLS2, SPRYD4*	0.033	0.004	1.40E-18	77.4
rs1047891	A	C	*CPS1*	−0.029	0.003	1.25E-20	86.7
rs10750864	T	A	*SLC43A1*	−0.055	0.003	3.37E-59	263.2
rs10826337	A	G	*SLC16A9, MRPL50P4*	0.017	0.003	1.30E-08	32.3
rs117040573	A	G	*C12orf42*	0.037	0.006	3.92E-10	39.2
rs12830698	G	T	*STAB2*	0.115	0.014	6.48E-17	69.8
rs13254494	C	T	*SLC25A37*	−0.018	0.003	1.02E-09	37.3
rs140584594	A	G	*GSTM1*	0.022	0.003	6.40E-12	47.2
rs1522298	C	G	*PAH*	−0.06	0.003	7.19E-85	381.1
rs17253619	C	T	*WDHD1*	0.054	0.004	1.45E-33	145.8
rs1800759	T	G	*ADH4, LOC100507053*	0.018	0.003	7.58E-10	37.9
rs2239328	T	C	*ABCC6*	−0.026	0.003	2.29E-16	67.3
rs34121855	G	T	*MLXIPL*	−0.021	0.004	1.04E-08	32.8
rs3757132	T	C	*SLC17A1*	−0.036	0.003	1.83E-27	117.9
rs61935426	A	C	*LINC02456*	0.086	0.012	9.04E-13	51
rs73063122	C	A	*SLCO1B1*	−0.029	0.004	2.36E-13	53.7
rs75017413	A	T	*SLC38A4*	0.041	0.006	2.41E-12	49.1
rs870072	C	T	*PAH*	0.111	0.003	2.66E-303	1408.7
rs932316	C	T	*SCGN, CARMIL1*	−0.021	0.004	1.44E-08	32.1
rs99780	T	C	*FADS2*	0.02	0.003	9.88E-11	41.8

Supplementary Table 6. Genetic instruments for tyrosine in the overall analysis.

SNP	effect_allele	other_allele	gene	beta	se	*p*-value	F-statistics
rs10027275	G	C	*ARHGAP10*	0.032	0.003	4.72E-22	93.2
rs10164853	G	A	*ACVR1C*	0.036	0.006	1.27E-10	41.3
rs10217762	C	T	*CDKN2B-AS1*	−0.019	0.003	9.61E-11	41.9
rs1043011	T	G	*GLS2, SPRYD4*	0.065	0.004	3.94E-69	308.8
rs10750864	T	A	*SLC43A1*	−0.02	0.003	2.06E-09	35.9
rs11263465	G	A	*LOC105369370*	0.027	0.004	1.86E-10	40.6
rs114232169	T	G	*HRG, HRG-AS1*	0.025	0.003	1.88E-16	67.7

rs11614623	T	C	*HPD*	0.049	0.004	4.14E-29	125.4
rs11643623	T	C	*ZNF276*	0.018	0.003	1.86E-08	31.6
rs11706810	C	T	*TRIM59, TRIM59-IFT80*	−0.02	0.003	4.93E-12	47.7
rs12212085	C	A	*SLC16A10*	0.171	0.004	0	1699.8
rs123698	G	C	*PTBP1*	0.018	0.003	9.64E-10	37.4
rs12596084	C	A	*RNA5SP427, MPHOSPH10P1*	−0.02	0.003	4.99E-10	38.7
rs12811045	G	A	*LOC102723639*	0.023	0.004	6.24E-11	42.7
rs12824518	T	C	/	0.028	0.004	4.60E-11	43.3
rs13107325	T	C	*SLC39A8*	−0.037	0.005	2.36E-11	44.6
rs13142887	T	A	/	−0.018	0.003	7.40E-09	33.4
rs13281892	G	A	*SLC7A2*	−0.021	0.003	2.55E-11	44.5
rs1345901	C	T	*LOC105371356*	0.022	0.003	1.15E-13	55.1
rs140584594	A	G	*GSTM1*	0.019	0.003	4.71E-09	34.3
rs1433210	C	A	*LINC01091*	−0.02	0.003	5.78E-09	33.9
rs150851429	C	G	*LOC105371334*	0.376	0.013	5.51E-198	901.1
rs151175127	T	C	/	−0.059	0.007	4.22E-17	70.7
rs1531022	A	G	*UGT2B15*	0.018	0.003	6.05E-10	38.3
rs17050272	A	G	*LOC105373585*	0.023	0.003	7.05E-15	60.6
rs174537	T	G	*MYRF*	0.036	0.003	5.38E-32	138.6
rs1800961	T	C	*HNF4A*	−0.084	0.008	4.67E-24	102.3
rs183657985	T	C	*EXOC3L2*	0.021	0.003	4.65E-10	38.8
rs1883711	C	G	*LINC01370, MAFB*	0.067	0.009	6.17E-15	60.8
rs194742	T	C	*MAGOH3P, ZFP36L1*	−0.024	0.004	7.99E-10	37.8
rs204926	G	A	*LMO1*	0.017	0.003	3.09E-09	35.1
rs2126263	G	A	*LOC157273*	0.034	0.005	1.13E-12	50.6
rs2189966	C	T	*JAZF1*	−0.037	0.004	3.74E-25	107.3
rs2393775	G	A	*HNF1A*	0.052	0.003	2.32E-69	309.9
rs28601761	G	C	*TRIB1AL*	−0.044	0.003	4.79E-49	216.7
rs34396849	C	A	*PGBD1*	0.031	0.004	1.80E-12	49.7
rs35048664	G	T	*PAH*	0.02	0.003	5.65E-10	38.4
rs35757209	T	C	*UNK*	0.024	0.003	1.42E-15	63.7
rs41289886	A	G	*RPF2*	0.066	0.012	1.88E-08	31.6
rs4416405	A	G	/	0.016	0.003	3.95E-08	30.2
rs4493565	A	C	*SHROOM3*	0.016	0.003	1.40E-08	32.2
rs4665972	T	C	*SNX17*	−0.022	0.003	7.13E-14	56
rs4722551	C	T	*LOC105375199*	−0.023	0.004	3.18E-09	35.1
rs4921914	C	T	*PSD3, NAT2*	−0.026	0.003	2.30E-14	58.3
rs511154	A	G	*RPL31P23, PCCB*	−0.037	0.003	1.24E-27	118.7
rs529565	C	T	*ABO*	0.019	0.003	1.50E-09	36.5
rs56058728	A	G	*INSR*	0.021	0.004	1.03E-08	32.8
rs56337219	T	C	*SLC16A10*	−0.066	0.007	3.57E-21	89.2
rs56401710	C	A	*SLC22A7,*	0.024	0.003	3.35E-16	66.6

			LOC124901319				
rs60718363	C	T	*WWC2*	0.02	0.003	8.86E-09	33.1
rs61676179	A	C	*DEPDC5*	−0.046	0.007	1.88E-12	49.6
rs62062797	G	T	*MAPT*	−0.019	0.003	2.44E-08	31.1
rs62466318	T	C	*MLXIPL*	−0.027	0.004	3.27E-14	57.6
rs6575900	C	G	*WDR20, LOC105370677*	−0.036	0.004	6.02E-23	97.3
rs6754311	C	T	*DARS1*	−0.021	0.003	2.01E-09	36
rs6831352	T	C	*ADH4, LOC100507053*	0.045	0.003	4.21E-46	203.2
rs6906327	A	G	*CDKAL1*	0.017	0.003	2.17E-08	31.3
rs715	C	T	*CPS1*	−0.026	0.003	3.28E-17	71.2
rs73079476	C	A	*SLCO1B1*	−0.053	0.004	1.67E-38	168.4
rs73158176	G	C	*PRKAG2*	−0.019	0.003	2.24E-08	31.3
rs738408	T	C	*PNPLA3*	0.025	0.003	8.18E-13	51.2
rs7404381	A	G	*AP1G1*	−0.052	0.003	4.54E-67	299.4
rs7537281	T	A	*PPIAP34, ZBTB40*	−0.024	0.004	1.12E-10	41.6
rs75891099	A	C	*REV3L*	0.154	0.02	3.78E-15	61.8
rs77042499	C	T	*TRAF3IP2-AS1*	0.089	0.01	2.69E-17	71.6
rs78424108	G	C	*WBP4, MIR3168*	−0.034	0.006	3.89E-10	39.2
rs78802502	A	G	*SLC38A4*	0.082	0.006	1.07E-45	201.3
rs7909960	A	T	*JMJD1C*	0.038	0.003	5.61E-39	170.6
rs79687284	C	G	*PROX1-AS1*	0.051	0.008	1.38E-10	41.2
rs8021303	A	G	*WDHD1*	−0.019	0.003	1.42E-10	41.1
rs8100204	A	G	*SUGP1*	0.029	0.004	4.64E-12	47.8
rs8122094	G	C	*TOP1*	0.019	0.003	4.37E-08	30
rs814573	T	A	*APOC1P1, APOC1*	−0.029	0.004	1.41E-14	59.2
rs9972653	T	G	*FTO*	0.019	0.003	1.81E-10	40.7

Supplementary Table 7. Genetic instruments for phenylalanine in the sex-specific analysis.

SNP	effect_allele	other_allele	gene	beta	se	*p*-value	F-statistics
Male							
rs1321250	C	T	/	−0.036	0.005	3.46E-11	43.9
rs3757132	T	C	*SLC17A1*	−0.038	0.005	9.59E-15	60.0
rs1009062	G	T	*GSTA2*	−0.025	0.004	2.08E-08	31.4
rs10750864	T	A	*SLC43A1*	−0.063	0.005	3.54E-37	162.3
rs75918019	G	A	*SLC38A4*	0.056	0.009	3.89E-09	34.7
rs76169231	C	T	*LINC02456*	−0.032	0.005	1.44E-09	36.6
rs1718292	G	A	*PAH*	0.115	0.004	3.15E-152	690.7
rs1498691	G	A	*PAH*	−0.060	0.005	1.28E-39	173.5
rs12367892	C	G	*C12orf42*	0.088	0.015	1.76E-09	36.2
rs17253619	C	T	*WDHD1*	0.062	0.007	1.72E-21	90.6
rs2239327	A	C	*ABCC6*	−0.031	0.005	7.45E-11	42.4
rs2229742	C	G	*NRIP1*	0.043	0.007	8.81E-10	37.6

Female							
rs1047891	A	C	*CPS1*	−0.039	0.004	1.13E-19	82.4
rs1408268	A	T	*SLC17A1*	−0.035	0.005	1.08E-14	59.7
rs10750864	T	A	*SLC43A1*	−0.048	0.005	2.24E-25	108.4
rs4149058	G	A	*SLCO1B1*	−0.026	0.005	4.82E-08	29.8
rs2694917	C	T	*RBMS2*	0.037	0.005	1.69E-13	54.3
rs1498694	A	G	*PAH, LOC124902999*	0.111	0.004	2.08E-164	746.7
rs9804734	T	C	*PAH*	−0.061	0.004	1.97E-47	209.3
rs12830698	G	T	*STAB2*	0.114	0.019	1.36E-09	36.7
rs17253619	C	T	*WDHD1*	0.047	0.006	7.39E-15	60.5
rs2239328	T	C	*ABCC6*	−0.024	0.004	4.38E-08	30.0

Supplementary Table 8. Genetic instruments for tyrosine in the sex-specific analysis.

SNP	effect_allele	other_allele	gene	beta	se	*p*-value	F-statistics
Male							
rs17050272	A	G	*LOC105373585*	0.025	0.004	4.28E-09	34.5
rs12614487	T	C	*ACVR1C*	0.047	0.008	5.43E-09	34.0
rs511154	A	G	*RPL31P23, PCCB*	−0.037	0.005	1.35E-13	54.8
rs12632030	C	T	*SMC4, TRIM59-IFT80*	−0.024	0.004	2.28E-08	31.2
rs5402	A	T	*SLC2A2*	0.040	0.007	1.27E-09	36.9
rs114232169	T	G	*HRG, HRG-AS1*	0.031	0.005	8.50E-12	46.6
rs10020631	A	G	*TMPRSS11E*	0.031	0.005	4.99E-10	38.7
rs1531022	A	G	*UGT2B15*	0.030	0.004	2.93E-12	48.7
rs1800759	T	G	*ADH4, LOC100507053*	0.048	0.004	8.85E-28	119.3
rs13107325	T	C	*SLC39A8*	−0.044	0.008	3.65E-08	30.3
rs10027275	G	C	*ARHGAP10*	0.028	0.005	1.53E-08	32.0
rs72839445	A	G	*CDCA7P1, POM121L2*	0.038	0.007	3.03E-08	30.7
rs1051952	C	A	*TEAD3, TULP1, LOC124901309*	−0.025	0.004	3.79E-09	34.7
rs62646255	C	T	*SLC22A7*	0.029	0.004	4.19E-11	43.5
rs12206654	C	T	*MFSD4B-DT*	0.192	0.006	5.81E-215	979.3
rs17717962	C	T	*LOC105377944*	0.088	0.015	1.08E-08	32.7
rs35614968	A	G	*FYN*	−0.129	0.014	3.85E-21	89.0
rs116862171	G	A	*FYN*	0.100	0.017	6.55E-09	33.7
rs4719841	G	A	*LOC105375199*	0.027	0.004	7.84E-10	37.8
rs73091233	C	T	*JAZF1*	−0.085	0.010	1.83E-16	67.8
rs9987289	A	G	*LOC157273*	0.044	0.007	2.22E-09	35.8
rs2980888	T	C	*TRIB1AL*	0.061	0.005	6.00E-39	170.4
rs10761756	T	C	*JMJD1C*	0.049	0.004	1.00E-30	132.8
rs2297644	C	T	*HOGA1*	−0.031	0.006	4.63E-08	29.9
rs174537	T	G	*MYRF*	0.035	0.004	3.80E-15	61.8
rs4766214	G	A	*LOC105369612*	−0.025	0.004	4.88E-09	34.2
rs1871395	G	A	*SLCO1B1*	−0.059	0.006	1.07E-22	96.1
rs79295634	G	A	*SLC38A4*	0.086	0.009	1.82E-23	99.6

rs1043011	T	G	*GLS2, SPRYD4*	0.067	0.006	1.70E-34	150.0
rs12811045	G	A	*LOC102723639*	0.032	0.005	7.46E-10	37.9
rs2464190	C	T	*HNF1A*	0.047	0.004	1.27E-26	114.0
rs4760099	A	G	*HPD*	0.052	0.006	9.16E-16	64.6
rs78424108	G	C	*WBP4, MIR3168*	−0.047	0.008	1.19E-08	32.5
rs3783642	C	T	*GCH1*	−0.024	0.004	3.71E-08	30.3
rs6575900	C	G	*WDR20, LOC105370677*	−0.042	0.005	5.89E-15	60.9
rs9746832	A	G	*LOC105371334*	0.087	0.004	2.37E-92	415.5
rs116992380	C	T	*PKD1L3*	0.206	0.015	3.23E-45	199.1
rs8047723	T	G	/	0.029	0.004	7.98E-11	42.3
rs10221244	G	A	*UNK*	0.026	0.004	8.30E-09	33.2
rs123698	G	C	*PTBP1*	0.024	0.004	4.04E-08	30.1
rs58542926	T	C	*TM6SF2*	0.060	0.008	8.09E-14	55.8
rs814573	T	A	*APOC1P1, APOC1*	−0.032	0.006	1.18E-08	32.5
rs1883711	C	G	*LINC01370, MAFB*	0.078	0.013	9.02E-10	37.5
rs1800961	T	C	*HNF4A*	−0.085	0.012	3.17E-12	48.6
rs738408	T	C	*PNPLA3*	0.031	0.005	2.30E-09	35.7
Female							
rs4665972	T	C	*SNX17*	−0.025	0.004	1.11E-09	37.1
rs1047891	A	C	*CPS1*	−0.032	0.004	1.15E-14	59.6
rs895893	C	T	*RPL31P23, PCCB*	−0.037	0.005	1.12E-15	64.2
rs35491981	C	T	*LOC100507053*	0.045	0.004	2.94E-24	103.3
rs7697204	C	T	*ARHGAP10*	0.035	0.004	2.53E-15	62.6
rs35261542	A	C	*CDKAL1*	0.027	0.004	2.24E-09	35.8
rs11961853	T	C	*UBQLN1P1, MICC*	0.028	0.005	4.84E-08	29.8
rs75661418	G	A	*SLC16A10*	−0.076	0.012	9.66E-10	37.4
rs12206654	C	T	*MFSD4B-DT*	0.177	0.006	7.63E-218	992.5
rs56236906	A	G	*FYN*	0.083	0.014	5.28E-09	34.1
rs35614968	A	G	*FYN*	−0.109	0.012	1.11E-18	77.9
rs146133919	C	T	*FYN*	−0.076	0.013	4.92E-09	34.2
rs2189966	C	T	*JAZF1*	−0.034	0.005	1.79E-12	49.7
rs34346326	C	T	*MLXIPL*	−0.030	0.005	4.09E-10	39.1
rs330093	G	C	*PPP1R3B-DT*	0.026	0.005	2.61E-08	31.0
rs28601761	G	C	*TRIB1AL*	−0.037	0.004	3.83E-20	84.5
rs7902343	T	C	*JMJD1C*	0.029	0.004	1.83E-13	54.2
rs174555	C	T	*FADS1*	0.038	0.004	3.29E-19	80.3
rs4149059	T	C	*SLCO1B1*	−0.041	0.005	5.37E-18	74.7
rs76943648	C	T	*SLC38A4*	0.078	0.008	6.15E-24	101.8
rs2657879	G	A	*GLS2, SPRYD4*	0.064	0.005	2.31E-37	163.2
rs2393775	G	A	*HNF1A*	0.057	0.004	1.22E-45	201.1
rs372273603	G	A	*HPD*	0.047	0.006	5.04E-16	65.8
rs1595261	C	A	/	0.025	0.004	1.12E-08	32.6
rs6575900	C	G	*WDR20, LOC105370677*	−0.031	0.005	3.16E-10	39.6
rs150851429	C	G	*LOC105371334*	0.389	0.017	3.12E-119	539.0
rs11640725	A	G	*AP1G1, LOC124903714*	−0.046	0.004	9.96E-30	128.2

rs1135688	C	T	*UNC13D*	0.025	0.004	2.82E-09	35.3
rs1800961	T	C	*HNF4A*	−0.083	0.011	1.96E-13	54.0

Supplementary Table 9. The outliers detected in MR-PRESSO.

Phenotype	Sex	Outlier
Phenylalanine	Overall	rs73063122
	Female	rs1047891
Tyrosine	Overall	rs183657985, rs4493565

Supplementary Table 10. MR results after exclusion of SNPs significantly associated with potential confounders.

Phenotype	Sex	Method	beta	se	*p*-value
Phenylalanine	Overall	Inverse variance weighted	−0.05	0.43	0.90
		Weighted median	−0.20	0.37	0.58
		Weighted mode	−0.09	0.35	0.80
		MR-PRESSO	0.17	0.33	0.61
Phenylalanine	Male	Inverse variance weighted	0.76	0.31	0.01
		Weighted median	0.60	0.39	0.12
		Weighted mode	0.60	0.39	0.13
		MR-PRESSO	0.76	0.28	0.02
Phenylalanine	Female	Inverse variance weighted	−0.66	0.60	0.27
		Weighted median	−0.43	0.46	0.35
		Weighted mode	−0.39	0.46	0.40
		MR-PRESSO	−0.36	0.48	0.48
Tyrosine	Overall	Inverse variance weighted	−0.60	0.28	0.03
		Weighted median	−0.14	0.29	0.62
		Weighted mode	−0.15	0.28	0.59
		MR-PRESSO	−0.41	0.25	0.10
Tyrosine	Male	Inverse variance weighted	−0.69	0.32	0.03
		Weighted median	−0.47	0.34	0.16
		Weighted mode	−0.26	0.35	0.47
		MR-PRESSO	−0.69	0.32	0.04
Tyrosine	Female	Inverse variance weighted	−0.68	0.30	0.03
		Weighted median	−0.16	0.36	0.66
		Weighted mode	−0.21	0.33	0.53
		MR-PRESSO	−0.68	0.30	0.03

For phenylalanine, rs34121855 was excluded in the overall analysis, due to its potential pleiotropic effect concerning alcohol intake frequency. For tyrosine, the following SNPs were excluded due to their potential pleiotropic effects: in the overall analysis, rs6754311 and rs7909960 were excluded due to their associations with age completed full time education. Additionally, rs13107325, rs4665972, rs62062797, rs62466318, and rs9972653 were excluded because of their potential effects on alcohol intake frequency. Within the female subgroup, rs7902343 was excluded for its pleiotropic effect with the age of completing full-time education, and rs4665972 was also excluded due to its impact on alcohol intake frequency. In the male subgroup, rs13107325 was removed from the analysis for its pleiotropic effect on alcohol intake frequency.

Supplementary Table 11. Sensitivity analysis on the association of genetically predicted phenylalanine and tyrosine with life years using genetic instruments from a GWAS without UK Biobank participants.

Exposure	Sex	Method	#SNPs	Beta (95% CI)	*p*-value	outlier
Phenylalanine	Overall	Inverse variance weighted	9	0.35 (−0.74, 1.44)	0.53	
		Weighted median	9	−0.10 (−1.04, 0.85)	0.84	
		Weighted mode	9	−0.12 (−1.14, 0.90)	0.82	
		MR-PRESSO	8	−0.02 (−1.23, 1.20)	0.97	rs28365897
	Male	Inverse variance weighted	9	1.05 (0.03, 2.08)	0.04	
		Weighted median	9	1.00 (0.05, 1.95)	0.04	
		Weighted mode	9	0.94 (−0.06, 1.93)	0.07	
		MR-PRESSO	9	1.05 (−0.15, 2.26)	0.08	
	Female	Inverse variance weighted	9	−0.12 (−1.55, 1.32)	0.88	
		Weighted median	9	−0.19 (−1.31, 0.94)	0.74	
		Weighted mode	9	−0.39 (−1.60, 0.82)	0.53	
		MR-PRESSO	7	−0.19 (−1.09, 0.71)	0.63	rs1047891, rs28365897
Tyrosine	Overall	Inverse variance weighted	19	−0.21 (−2.16, 1.73)	0.83	
		Weighted median	19	−0.31 (−0.90, 0.28)	0.30	
		Weighted mode	19	−0.36 (−0.89, 0.17)	0.19	
		MR-PRESSO	17	−0.67 (−1.24, −0.09)	0.03	rs4149083, rs429358
	Male	Inverse variance weighted	19	−0.52 (−2.03, 0.99)	0.50	
		Weighted median	19	−0.47 (−1.13, 0.19)	0.16	
		Weighted mode	19	−0.47 (−1.09, 0.15)	0.13	
		MR-PRESSO	17	−0.82 (−1.67, 0.03)	0.06	rs1021956, rs117866491
	Female	Inverse variance weighted	19	0.09 (−2.20, 2.38)	0.94	
		Weighted median	19	−0.14 (−0.88, 0.59)	0.70	
		Weighted mode	19	−0.18 (−0.86, 0.50)	0.61	
		MR-PRESSO	17	−0.58 (−1.18, 0.02)	0.06	rs1021956, rs117866491

Supplementary Table 12. Power calculation in the MR analysis on lifespan.

Exposure	Sex	r^2	Case	Control	Sample size	Effect size detected (odds ratio)	Effect size detected (life years)
Phenylalanine	Overall	0.01	208,118	181,048	389,166	1.09	−1.72
	Men		317,652	97,659	415,311	1.10	−2.18
	Women		246,941	165,996	412,937	1.09	−2.23
Tyrosine	Overall	0.03	208,118	181,048	389,166	1.06	−1.17
	Men		317,652	97,659	415,311	1.06	−1.33
	Women		246,941	165,996	412,937	1.06	−1.51

The calculation of r^2 was based on $beta^2 \times 2 \times (EAF) \times (1\text{-}EAF)$. The number of cases and controls are from the GWAS of parental lifespan we used in the MR analysis (Pilling LC et al., Aging (Albany NY). 2017; 9:2504–20). The power calculations for the MR analysis were performed using the online tool available at https://sb452.shinyapps.io/power/.

Supplementary Table 13. Associations of genetic instruments for phenylalanine with potential confounders in UK Biobank.

SNP	*p*-value with potential confounders					
	Townsend deprivation index at recruitment	Age completed full time education	Current tobacco smoking	Alcohol intake frequency	Time spent doing moderate physical activity	Time spent doing vigorous physical activity
Overall						
rs1009062	4.08E-01	1.48E-01	7.22E-01	3.80E-01	9.17E-01	6.08E-01
rs1043011	2.76E-01	2.26E-01	3.28E-01	3.60E-02	1.99E-01	3.57E-01
rs1047891	7.47E-01	5.65E-01	7.13E-01	6.55E-01	6.43E-01	5.96E-01
rs10750864	8.60E-01	7.38E-01	1.92E-01	5.31E-01	2.82E-01	8.53E-01
rs10826337	8.67E-01	2.28E-01	3.65E-01	8.92E-01	9.91E-01	6.33E-01
rs117040573	6.06E-01	3.96E-01	3.23E-01	7.11E-01	9.31E-01	8.96E-01
rs12830698	1.07E-01	7.87E-01	5.99E-01	2.05E-01	3.95E-01	9.10E-01
rs13254494	3.63E-02	9.43E-01	2.52E-01	2.56E-01	3.23E-01	5.61E-01
rs1522298	9.87E-01	2.09E-01	9.15E-01	5.10E-01	2.26E-01	5.16E-01
rs17253619	2.55E-01	7.67E-01	7.82E-01	3.43E-01	8.67E-01	7.11E-02
rs1800759	9.50E-02	1.58E-01	1.07E-02	6.18E-02	9.67E-01	4.28E-01
rs2239328	1.78E-01	4.65E-02	4.32E-01	2.89E-01	3.50E-01	5.83E-01
rs34121855	9.48E-01	1.34E-01	4.78E-01	1.45E-09	3.96E-01	7.49E-01
rs3757132	6.20E-02	1.97E-01	9.95E-01	2.89E-01	8.80E-01	1.35E-01
rs61935426	2.01E-01	6.92E-01	1.86E-01	5.99E-01	9.95E-01	9.35E-01
rs73063122	9.59E-01	3.35E-01	7.93E-01	6.59E-03	3.12E-01	3.17E-01
rs75017413	6.30E-01	4.46E-01	2.50E-01	4.75E-01	7.10E-01	6.04E-02
rs870072	6.17E-01	5.12E-01	6.58E-02	7.36E-01	3.44E-01	1.80E-01
rs932316	4.21E-02	6.04E-01	7.37E-01	4.04E-02	5.17E-01	8.98E-01
rs99780	5.22E-03	2.17E-01	1.33E-01	2.42E-01	2.80E-01	3.63E-01
Male						
rs1009062	6.84E-01	3.99E-02	2.48E-01	2.78E-01	7.57E-01	5.05E-01
rs10750864	9.38E-01	2.97E-01	7.82E-01	9.46E-01	2.74E-01	2.26E-01
rs12367892	7.20E-01	7.03E-01	7.30E-01	8.41E-01	6.71E-03	6.59E-01
rs1321250	1.42E-01	9.64E-01	3.54E-01	3.01E-01	6.76E-01	1.55E-01
rs1498691	6.95E-01	9.02E-01	2.29E-01	8.08E-01	6.10E-01	7.84E-01
rs1718292	1.53E-01	7.76E-01	6.69E-01	3.65E-01	9.39E-01	3.96E-02
rs17253619	7.90E-02	6.80E-01	7.19E-01	2.55E-01	2.90E-01	6.11E-02
rs2229742	8.04E-01	7.03E-01	3.07E-01	3.47E-02	4.54E-02	7.23E-01
rs2239327	1.82E-01	3.12E-02	8.02E-01	6.36E-03	1.03E-01	9.26E-01
rs3757132	2.21E-01	7.37E-01	5.54E-02	4.99E-01	5.85E-01	4.30E-01
rs75918019	5.99E-01	8.97E-01	1.45E-01	5.11E-01	3.82E-01	1.69E-02
rs76169231	6.03E-01	2.98E-01	6.38E-01	6.11E-01	4.32E-01	2.08E-01
Female						
rs1047891	5.59E-01	7.24E-01	6.97E-01	8.12E-01	4.00E-01	4.00E-01
rs10750864	9.58E-01	3.27E-01	4.78E-01	3.22E-01	1.93E-01	1.93E-01
rs12830698	7.16E-02	5.36E-01	8.82E-01	2.82E-01	7.36E-02	7.36E-02
rs1408268	8.21E-01	3.74E-01	1.10E-01	6.44E-02	9.39E-02	9.39E-02
rs1498694	8.27E-01	6.17E-01	3.80E-02	2.02E-01	7.24E-01	7.24E-01

rs17253619	4.36E-01	6.12E-01	3.71E-01	2.35E-01	5.94E-01	5.94E-01
rs2239328	9.82E-01	8.96E-01	9.93E-01	4.51E-01	9.77E-01	9.77E-01
rs2694917	3.31E-01	4.91E-01	3.02E-01	1.50E-01	6.98E-01	6.98E-01
rs4149058	8.92E-01	2.97E-01	4.81E-01	5.96E-01	8.76E-01	8.76E-01
rs9804734	6.82E-01	9.49E-02	8.91E-02	6.57E-01	2.65E-01	2.65E-01

A significant association was observed between rs34121855 and alcohol intake frequency in the overall analysis.

Supplementary Table 14. Associations of genetic instruments for tyrosine with potential confounders in UK Biobank.

SNP	*p*-value with potential confounders					
	Townsend deprivation index at recruitment	Age completed full time education	Current tobacco smoking	Alcohol intake frequency	Time spent doing moderate physical activity	Time spent doing vigorous physical activity
Overall						
rs10027275	4.22E-01	2.30E-02	5.63E-01	8.65E-01	7.62E-01	8.64E-01
rs10164853	5.43E-03	1.33E-01	4.75E-01	7.63E-01	3.17E-01	3.54E-01
rs10217762	1.98E-02	3.76E-03	2.82E-01	1.34E-01	6.48E-01	3.39E-01
rs1043011	2.76E-01	2.26E-01	3.28E-01	3.60E-02	1.99E-01	3.57E-01
rs10750864	8.60E-01	7.38E-01	1.92E-01	5.31E-01	2.82E-01	8.53E-01
rs11263465	9.19E-01	2.89E-01	6.34E-01	1.77E-01	6.03E-01	9.25E-02
rs11614623	7.33E-01	9.79E-01	1.47E-01	1.55E-01	5.68E-01	1.91E-01
rs11643623	7.83E-01	5.02E-01	3.52E-01	1.53E-01	2.93E-01	9.28E-01
rs11706810	2.01E-02	2.29E-02	3.01E-01	1.15E-04	3.83E-01	2.37E-01
rs12212085	6.01E-02	2.20E-02	1.25E-02	6.70E-01	7.28E-01	3.05E-01
rs123698	4.82E-02	8.98E-01	6.81E-01	7.04E-01	4.14E-01	4.17E-01
rs12596084	8.09E-01	8.16E-01	6.93E-01	3.84E-01	4.11E-01	3.00E-01
rs12811045	5.26E-01	6.75E-02	4.21E-01	6.74E-01	4.82E-01	6.15E-01
rs12824518	2.92E-01	8.89E-02	7.39E-01	8.43E-01	4.06E-02	6.61E-02
rs13107325	1.13E-01	6.86E-04	5.26E-05	6.90E-15	7.56E-02	6.82E-02
rs13142887	3.37E-01	1.24E-01	1.41E-01	8.19E-01	3.70E-02	9.90E-01
rs13281892	3.58E-01	9.48E-01	2.48E-01	8.42E-01	3.79E-01	4.60E-01
rs1345901	5.22E-01	5.33E-01	4.90E-01	1.19E-01	9.58E-01	5.72E-01
rs1433210	1.73E-01	5.45E-01	1.10E-01	4.10E-02	7.25E-01	7.86E-01
rs150851429	1.31E-01	5.11E-04	4.15E-01	3.92E-01	8.74E-01	8.21E-01
rs151175127	7.05E-01	4.91E-02	4.26E-01	2.07E-01	1.45E-01	1.39E-01
rs1531022	7.35E-01	9.55E-01	3.23E-01	1.98E-01	6.48E-01	6.56E-01
rs17050272	3.54E-01	9.43E-01	9.45E-02	8.25E-01	3.47E-02	2.13E-01
rs174537	1.53E-02	1.87E-01	1.74E-01	1.45E-01	4.00E-01	4.30E-01
rs1800961	2.23E-01	5.28E-01	3.89E-01	7.53E-01	8.20E-01	4.26E-01
rs183657985	5.15E-03	1.46E-04	4.42E-01	4.37E-04	7.74E-01	9.44E-01
rs1883711	5.45E-02	1.29E-01	3.08E-01	2.49E-02	9.61E-01	4.84E-01
rs194742	6.59E-01	5.53E-01	6.49E-01	6.10E-01	5.21E-01	7.71E-01
rs204926	5.16E-01	8.86E-01	7.45E-02	3.28E-01	8.67E-01	8.46E-01
rs2126263	3.92E-01	8.37E-01	9.10E-03	4.80E-03	7.82E-01	9.72E-01
rs2189966	5.23E-01	9.57E-01	2.26E-01	6.22E-02	1.06E-01	8.13E-01
rs2393775	4.72E-01	5.63E-01	5.15E-01	2.70E-02	7.19E-01	1.17E-01
rs28601761	1.47E-01	5.07E-02	9.22E-01	3.64E-05	3.42E-01	5.10E-01

rs34396849	5.75E-05	7.95E-02	6.57E-04	3.44E-01	9.10E-01	1.08E-01
rs35048664	1.00E+00	2.07E-01	9.25E-01	4.98E-01	2.25E-01	5.17E-01
rs35757209	9.56E-01	3.71E-01	8.82E-01	6.63E-01	8.48E-01	3.00E-02
rs41289886	1.80E-01	1.57E-01	5.29E-01	6.71E-01	2.04E-01	6.73E-01
rs4493565	1.80E-02	1.37E-01	4.81E-02	3.12E-02	7.66E-01	7.26E-01
rs4665972	3.83E-01	3.05E-01	1.74E-01	3.10E-55	3.41E-01	9.50E-01
rs4722551	7.84E-01	7.26E-01	3.27E-01	1.79E-02	8.01E-01	9.23E-02
rs4921914	9.65E-01	1.65E-04	5.17E-01	2.00E-01	9.49E-01	5.61E-01
rs511154	9.79E-01	2.48E-01	7.19E-01	2.46E-01	4.72E-01	7.69E-01
rs56058728	1.33E-01	4.31E-01	2.80E-01	2.33E-01	9.39E-01	6.63E-01
rs56337219	1.69E-01	3.95E-01	2.02E-01	3.05E-02	2.93E-01	1.53E-01
rs56401710	7.95E-01	7.13E-01	1.00E-01	1.26E-04	2.44E-01	9.62E-01
rs61676179	2.82E-01	6.06E-01	5.49E-01	6.93E-01	1.15E-01	5.39E-01
rs62062797	9.63E-01	2.27E-01	1.94E-02	3.09E-11	1.54E-01	3.53E-03
rs62466318	9.36E-01	2.10E-02	2.30E-01	1.44E-11	7.15E-01	8.15E-01
rs6575900	1.56E-01	4.02E-01	3.67E-01	4.12E-01	1.03E-03	3.14E-01
rs6754311	2.02E-01	2.11E-10	5.81E-01	7.68E-02	2.42E-01	1.30E-01
rs6831352	5.83E-02	9.62E-02	7.61E-04	8.25E-04	7.86E-01	5.56E-01
rs6906327	2.03E-01	8.49E-01	7.99E-01	7.48E-01	3.71E-01	9.83E-01
rs715	6.32E-01	6.41E-01	6.19E-01	6.64E-01	7.13E-01	7.73E-01
rs73079476	9.36E-01	3.13E-01	6.90E-01	1.80E-03	3.06E-01	2.31E-01
rs73158176	7.99E-03	8.07E-01	9.87E-01	4.71E-01	7.11E-01	7.25E-01
rs738408	7.65E-03	3.17E-01	6.52E-01	1.63E-02	4.29E-01	9.96E-01
rs7404381	4.49E-03	2.11E-02	5.01E-04	3.45E-01	1.56E-01	6.48E-01
rs7537281	2.43E-01	7.68E-02	3.64E-01	6.54E-03	2.69E-01	2.60E-01
rs77042499	1.92E-02	7.94E-02	5.66E-03	6.52E-01	3.56E-01	9.88E-01
rs78424108	9.02E-02	6.01E-01	4.25E-01	4.56E-01	1.57E-01	6.24E-01
rs78802502	6.19E-01	4.46E-01	2.39E-01	4.61E-01	7.62E-01	6.27E-02
rs7909960	2.69E-01	3.71E-11	1.34E-05	1.80E-01	5.50E-01	2.47E-01
rs79687284	4.32E-02	5.59E-01	1.74E-01	4.12E-01	2.23E-01	7.56E-02
rs8021303	1.93E-01	8.29E-01	1.59E-01	8.30E-01	2.03E-01	1.09E-01
rs8122094	4.36E-01	9.08E-01	9.73E-01	1.64E-04	9.24E-01	7.71E-01
rs9972653	5.78E-01	1.33E-01	5.42E-01	1.83E-09	9.07E-01	7.24E-01
Male						
rs10020631	3.37E-01	6.33E-01	2.86E-01	9.27E-01	9.18E-01	5.90E-01
rs10027275	6.33E-01	7.72E-01	2.10E-01	5.41E-02	7.83E-01	5.70E-01
rs10221244	8.65E-01	6.50E-01	2.53E-01	7.39E-01	3.85E-01	1.86E-01
rs1043011	2.09E-01	5.84E-01	5.85E-01	3.60E-01	1.53E-01	4.58E-01
rs1051952	4.67E-01	9.91E-01	1.05E-02	8.31E-01	1.08E-01	4.11E-01
rs10761756	1.37E-01	5.50E-03	9.17E-02	2.71E-01	3.41E-01	2.41E-01
rs116862171	9.54E-01	1.86E-01	2.23E-01	1.72E-02	3.13E-01	7.77E-02
rs116992380	7.85E-01	2.00E-01	6.55E-01	6.78E-01	5.73E-01	9.88E-01
rs12206654	1.08E-02	6.04E-01	1.46E-04	6.69E-01	8.37E-01	6.78E-01
rs123698	6.77E-01	7.31E-01	7.08E-01	2.12E-01	7.47E-01	7.23E-01
rs12614487	1.39E-01	4.70E-01	9.49E-01	4.17E-01	3.60E-01	1.29E-01
rs12632030	1.39E-01	5.51E-02	5.16E-02	1.23E-03	7.97E-01	2.88E-01
rs12811045	7.47E-01	6.10E-02	4.41E-01	2.17E-01	2.18E-01	7.27E-01
rs13107325	1.68E-01	3.25E-03	4.93E-02	5.49E-12	7.76E-01	4.58E-02

rs1531022	6.14E-01	9.52E-01	9.80E-01	2.29E-01	6.26E-01	7.28E-01
rs17050272	9.63E-01	1.29E-01	2.29E-01	5.13E-01	4.13E-01	7.76E-01
rs174537	3.09E-02	6.32E-02	2.29E-01	1.62E-01	3.31E-01	4.46E-01
rs17717962	8.91E-01	1.69E-01	3.42E-01	7.57E-01	7.63E-02	8.59E-01
rs1800759	2.33E-01	2.35E-02	3.49E-03	2.71E-01	2.18E-01	3.56E-01
rs1800961	1.49E-01	6.07E-01	4.53E-01	2.29E-01	3.94E-01	9.91E-01
rs1871395	3.06E-01	3.74E-01	3.22E-01	1.24E-01	6.88E-01	6.84E-01
rs1883711	4.79E-01	9.51E-01	8.20E-01	5.26E-03	9.49E-01	7.16E-01
rs2297644	3.71E-01	1.88E-01	4.44E-01	5.80E-01	1.45E-01	9.89E-01
rs2464190	7.12E-02	5.88E-01	8.99E-01	3.72E-01	8.19E-01	7.08E-01
rs2980888	3.53E-01	4.77E-01	1.13E-01	2.46E-02	1.79E-01	8.17E-01
rs35614968	9.48E-01	9.16E-01	8.74E-02	6.75E-01	8.01E-01	7.76E-01
rs3783642	7.39E-01	8.06E-01	1.69E-02	1.91E-01	1.90E-01	7.80E-02
rs4719841	7.44E-01	9.17E-01	7.96E-01	1.51E-01	9.87E-01	7.10E-02
rs4760099	4.16E-01	1.29E-01	8.34E-01	6.45E-01	4.76E-01	7.44E-02
rs4766214	8.23E-02	4.26E-01	6.95E-01	1.99E-01	6.21E-01	5.22E-02
rs511154	1.02E-01	2.72E-01	3.15E-01	2.24E-01	6.74E-01	3.41E-01
rs58542926	5.20E-01	1.85E-03	5.12E-01	3.45E-01	2.19E-01	8.95E-01
rs62646255	3.53E-01	7.26E-01	5.09E-01	6.42E-04	6.09E-01	2.12E-01
rs6575900	4.22E-01	2.10E-01	3.69E-01	3.43E-01	2.32E-04	1.68E-01
rs72839445	3.77E-03	5.10E-01	8.81E-05	9.95E-01	6.33E-01	8.53E-01
rs73091233	4.76E-01	1.37E-01	4.59E-01	2.39E-01	6.13E-01	4.65E-01
rs738408	4.06E-02	6.35E-01	1.10E-01	3.69E-01	8.91E-02	1.40E-01
rs78424108	2.31E-03	9.21E-01	3.82E-01	2.43E-01	6.69E-01	5.03E-01
rs79295634	4.18E-01	7.96E-01	1.29E-01	9.06E-01	7.53E-02	1.96E-03
rs8047723	7.49E-01	1.66E-01	1.35E-01	8.25E-01	8.57E-01	2.34E-01
rs9987289	9.13E-01	6.10E-02	2.62E-02	7.12E-01	1.41E-01	8.95E-01
Female						
rs1047891	5.59E-01	7.24E-01	6.97E-01	8.12E-01	4.00E-01	4.00E-01
rs1135688	4.84E-01	4.03E-01	6.07E-01	3.80E-01	9.52E-01	9.52E-01
rs11640725	2.40E-02	1.90E-03	1.66E-01	4.00E-01	8.47E-01	8.47E-01
rs12206654	7.67E-01	3.85E-01	4.97E-01	3.03E-01	4.35E-01	4.35E-01
rs146133919	9.49E-01	3.07E-01	6.54E-01	2.73E-01	2.37E-01	2.37E-01
rs150851429	9.94E-01	2.08E-03	8.76E-01	1.73E-01	6.60E-01	6.60E-01
rs1595261	4.71E-01	7.72E-01	9.28E-01	6.24E-01	9.97E-01	9.97E-01
rs174555	5.08E-03	7.43E-01	5.62E-01	5.13E-01	2.75E-01	2.75E-01
rs1800961	8.75E-01	9.62E-01	3.47E-01	7.43E-01	4.93E-01	4.93E-01
rs2189966	1.11E-01	6.17E-01	8.64E-01	6.47E-01	7.46E-01	7.46E-01
rs2393775	1.24E-01	8.55E-01	5.90E-02	2.07E-01	1.59E-02	1.59E-02
rs2657879	2.39E-01	1.53E-01	5.06E-01	1.94E-01	3.68E-01	3.68E-01
rs28601761	1.90E-01	5.31E-02	1.72E-01	2.04E-03	3.65E-01	3.65E-01
rs330093	7.26E-01	3.04E-03	9.60E-01	2.46E-01	1.45E-01	1.45E-01
rs34346326	3.56E-01	8.05E-01	3.10E-01	1.33E-05	9.21E-01	9.21E-01
rs35261542	3.39E-01	4.68E-01	3.70E-01	1.09E-01	1.19E-01	1.19E-01
rs35491981	4.99E-01	8.94E-01	2.43E-01	5.44E-01	5.20E-01	5.20E-01
rs35614968	4.62E-01	1.11E-01	4.70E-01	9.63E-01	2.42E-01	2.42E-01
rs372273603	6.15E-01	1.19E-01	2.54E-01	3.27E-01	7.76E-01	7.76E-01
rs4149059	9.03E-01	3.09E-01	5.11E-01	6.02E-01	8.72E-01	8.72E-01

rs4665972	7.50E-01	6.57E-01	3.71E-02	8.95E-21	7.45E-01	7.45E-01
rs56236906	5.05E-02	3.78E-01	1.28E-01	6.92E-01	8.54E-01	8.54E-01
rs6575900	7.37E-01	8.57E-01	7.02E-01	1.89E-01	4.91E-01	4.91E-01
rs75661418	9.41E-01	6.46E-01	2.00E-01	2.40E-01	4.78E-01	4.78E-01
rs76943648	9.59E-01	6.52E-01	4.48E-01	4.42E-01	7.13E-01	7.13E-01
rs7697204	3.62E-01	2.76E-01	3.39E-01	5.81E-01	4.35E-01	4.35E-01
rs7902343	2.70E-02	3.23E-09	2.27E-02	1.50E-01	9.07E-01	9.07E-01
rs895893	3.90E-01	9.92E-01	5.84E-01	3.69E-01	3.92E-01	3.92E-01
